# Risk-based evaluation of machine learning-based classification methods used for medical devices

**DOI:** 10.1186/s12911-025-02909-9

**Published:** 2025-03-11

**Authors:** Martin Haimerl, Christoph Reich

**Affiliations:** Furtwangen University of Applied Sciences, Furtwangen, Germany

**Keywords:** Classification, Risk management, Risk-based metrics, Decision theory, Medical devices

## Abstract

**Background:**

In the future, more medical devices will be based on machine learning (ML) methods. In general, the consideration of risks is a crucial aspect for evaluating medical devices. Accordingly, risks and their associated costs should be taken into account when assessing the performance of ML-based medical devices. This paper addresses the following three research questions towards a risk-based evaluation with a focus on ML-based classification models.

**Methods:**

First, we analyzed how often risk-based metrics are currently utilized in the context of ML-based classification models. This was performed using a literature research based on a sample of recent scientific publications. Second, we introduce an approach for evaluating such models where expected risks and associated costs are integrated into the corresponding performance metrics. Additionally, we analyze the impact of different risk ratios on the resulting overall performance. Third, we elaborate how such risk-based approaches relate to regulatory requirements in the field of medical devices. A set of use case scenarios were utilized to demonstrate necessities and practical implications, in this regard.

**Results:**

First, it was shown that currently most scientific publications do not include risk-based approaches for measuring performance. Second, it was demonstrated that risk-based considerations have a substantial impact on the outcome. The relative increase of the resulting overall risks can go up to 196% when the ratio between different types of risks (false negatives vs. false positives) changes by a factor of 10.0. Third, we elaborated that risk-based considerations need to be included into the assessment of ML-based medical devices, according to the relevant EU regulations and standards. In particular, this applies when a substantial impact on the clinical outcome / in terms of the risk-benefit relationship occurs.

**Conclusion:**

In summary, we demonstrated the necessity of a risk-based approach for the evaluation of medical devices which include ML-based classification methods. We showed that currently many scientific papers in this area do not include risk considerations. We developed basic steps towards a risk-based assessment of ML-based classifiers and elaborated consequences that could occur, when these steps are neglected. And, we demonstrated the consistency of our approach with current regulatory requirements in the EU.

**Supplementary Information:**

The online version contains supplementary material available at 10.1186/s12911-025-02909-9.

## Background

Machine learning (ML) is a revolutionary technology which is increasingly applied in medical applications, e.g. in radiology, pathology, or ophthalmology [1–3]. In specific tasks like diagnosis of diseases, e.g. skin cancer or retinal diseases, ML techniques achieve an equivalent or even better performance. Basically, this applies to accuracy rates in comparison with human experts [2, 4]. Such results indicate that the utilization of ML-based methods in actual clinical applications is promising. There already is a series of ML-based medical devices which were successfully placed on the market [5]. However, the clinical impact of the used devices has to be clearly demonstrated for the particular use case. For this purpose, a thorough evaluation with respect to the performance of the ML algorithms and their effect in the actual clinical environment has to be performed. For example, the requirements from the medical device regulation (MDR) [6] have to be fulfilled, before the device can be placed on the European Union (EU) market. In the future, also the proposed AI Act [7] has to be applied. The conformity with these regulations is usually proven by means of the harmonized standards associated with them. For performing risk management in the context of medical devices, the ISO 14971 [8] is the appropriate standard. Additionally, the technical report ISO/TR 24971 [9] provides more detailed guidance for the application of [8]. But, neither the MDR [6] nor [8, 9] contain specific information for ML-based devices. Recently, the technical report BSI/AAMI 34971 [10] was published, which was developed as a guide for applying the ISO 14971 to AI-/ML-based medical devices. This has the potential to close this gap. But, this also has some deficiencies. On the one hand, it is neither a harmonized standard for the MDR nor aligned with the AI Act. On the other hand, it focuses on the description of additional risks in the case of ML-based devices and does not include specific guidance for the consideration of risks within the evaluation of ML-based models and systems. Thus, a dedicated framework for addressing risk management in these cases is still missing.

The basic aim of the regulations is that the devices achieve a level of safety and performance which is appropriate for the clinical application. This includes a thorough analysis of potential risks and their associated impact as well as the clinical performance of the device with respect to the specific application and its context [6]. This does not only have to be applied during the validation, approval, or deployment phase of the device. According to the MDR and also ISO 14971, risk management has to be considered as an integral part of the entire development and product life cycle, starting from the early phases of product definition [6, 8]. According to this, risk assessment and management needs to be integrated into the development of an ML model, when it is intended to be used in a medical device.

In general, risk refers to an uncertain outcome which can occur during the application of the device. In particular, risks are related to potential harm and the term risk is defined as a combination of a certain likelihood, i.e. probability of occurrence, and a severity, i.e. magnitude of harm. This represents the definition in the MDR and ISO 14971 [6, 8]. Risk management is the process of systematically identifying, analyzing, assessing, and mitigating risks throughout the lifecycle of a product. This includes all development phases as well as the operation phase of the product. The central goal of risk management is to prevent harm to the patient or other users [8]. According to the MDR, risks have to be reduced as far as possible (ALARP principle) unless avoidance of further risk improvements does not have an adversarial effect on the risk-benefit relationship. Finally, the benefits have to outweigh the risks of the medical device [6]. Thus, it is crucial to evaluate the clinical outcome and demonstrate the clinical benefit of a device. In this regards, clinical benefit “means the positive impact of a device on the health of an individual, expressed in terms of a meaningful, measurable, patient-relevant clinical outcome(s), including outcome(s) related to diagnosis, or a positive impact on patient management or public health” [6].

For ML-based devices, this means that performance measures, which are utilized for the evaluation of the device, should be established which include such factors. This may include different phases in the development cycle. More specifically, this contains the development of the model with respect to the minimization of risks, the subsequent internal and external validation steps for the model as well as the final clinical validation of the entire device. The associated risks are one major component, when considering the clinical impact of the device. Additionally, the achieved benefits are important factors. In this context, risks and benefits should be considered as negative and positive components of the overall clinical outcome. Subsequently, this should be reflected in the performance measures. Pure accuracy rates, i.e. probabilities of errors, are not sufficient for evaluating the clinical performance of the device. In particular, the severity of errors and clinical impact of the model predictions have to be taken into account. Otherwise, the overall impact of the device cannot be addressed. Consequently, this leads to a potential violation of the ALARP principle for risk management [6, 8]. 

Currently, it seems that most scientific publications use standardized performance metrics, which basically focus on accuracy-based assessments to validate and test their ML models. This means that only the differences between the predicted results and the values from the reference data set (training, validation or test data sets) are compared. In these cases, a risk-based assessment is not included. For classification tasks and applications of supervised learning, standardized metrics refer to metrics like accuracy, precision, sensitivity/recall, $\:F1$ score, Matthews Correlation Coefficient ($\:MCC$), or Area under the $\:ROC$ Curve ($\:AUROC$) [11]. For example, this can be recognized in [12], where more than 70 medical image experts systematically analyzed requirements regarding the evaluation of machine learning models, e.g. for image-level classification tasks. But, only very limited references were included, where risks, costs, or benefits were included in the metrics. These exceptions refer to metrics in terms of net benefit [13] or expected costs [14]. Additionally, the weighted kappa statistic and the $\:{F}_{\beta\:}$ score were mentioned. These are specific performance metrics which include weighting factors which can be adapted to the particular application. More details about the definition of the metrics are provided later in Sect. Research question A – utilization of risk-based performance metrics in recent scientific publications. Concrete advices how to determine and integrate appropriate weights and thus to systematically adapt the outcome to the clinical impact were not given in [12]. Instead, most of the recommendations were based on the application of standardized metrics, like the ones mentioned above. The hypothesis that most recent scientific publications do not systematically address risk factors within the evaluation of ML models was one major goal of the analysis performed within this paper. The concrete formulation of this hypothesis / research question is provided at the end of this section.

In the mentioned standardized metrics, basically the number of errors is taken into account, when considering classification tasks. But, the clinical impact of the different type of errors is not considered [11, 12]. For example, a false negative (“missed diagnosis”) can have a substantially different clinical effect than a false positive (“false alarm”), when considering specific diagnostic applications. For example, a false positive within a cancer screening may have some harm (e.g. fear of potentially severe illness, additional tests or biopsies with potential harm). But, the harm in these cases is often considerably lower than the harm of false positives. A missed diagnosis may lead to substantial progression of the disease and eventually also to a lethal outcome [15]. These are important issues since the associated risk impact usually goes in contrary directions. Thus, the particular types of risks need to be balanced in a dedicated way.

The standard performance metrics, which are used in many publications [11, 12], do not include a dedicated assessment with regards to the risks and their clinical impact of a particular use case. Basically, accuracy rates, i.e. deviations between the training samples and the prediction of the models, are minimized. Implicitly, many performance metrics assume some kind of neutral situation, where a certain balancing of the relationship between false positives and false negatives is given. They do not intentionally weight different types of errors, but basically reflect the relationships as they are represented in the used data sets. For example, the same number of false positives and false negatives can be provided to achieve an equilibrium between both types of errors. In particular, this can be beneficial when one type of error is predominant [12]. In other cases, the data set may directly represent the prevalence of the disease. Defining appropriate relationships for the provided data set should be included as a part of the modeling / development phase of an ML model. Some error metrics like weighted kappa statistic and the $\:{F}_{\beta\:}$ score include a weighting between different types of errors. However, these approaches do not perform a dedicated integration of risk factors into the development of ML models. There are further important aspects which have to be considered in the quality management of ML-based medical devices, like data quality or uncertainty factors, e.g. in terms of confidence intervals for the results [16].

For utilization of ML-based techniques in medical devices, it has to be analyzed whether substantial improvements in terms of the risk-benefit relationship can be achieved, by means of appropriate risk mitigation measures. This is a central part of risk management and refers to the mentioned ALARP principle [6, 8]. Subsequently, the integration of risk factors like the disparate impact of false positives and false negatives have to be regarded. In particular, this is the case as long as this leads to substantial changes of the risk-benefit relationship. Otherwise, the reduction of risks and optimization of clinical benefits remains deficient. This does not only apply to evaluation steps, but also needs to be considered during the development phase [6, 8]. There may be different stages where this could be applied, e.g. integration of risk factors into the performance metrics for the training, validation, and testing of the ML-model, use-case specific adjustment of threshold parameters in the classification rule, or any other risk mitigation measure to reduce the probability and/or severity level of the risks.

Based on this rationale, the identification of the best model and its integration into a medical device should be performed in terms of the best decision not only with respect to measures of deviation. It should be addressed in terms of the best clinical outcome in combination with the strongest reduction of risks for the specific application. In general, an optimal balance needs to be found between benefits and risks [6, 8]. In this regard, risks can be considered as negative versions of benefits or in other words costs. More precisely, this refers to the harm which is associated with the particular risk [6, 8]. Subsequently, we use the term cost for a negative clinical impact which is measured in a quantitative way. Since the likelihood of risks and its corresponding harm is usually not given exactly, this can only be achieved in a probabilistic manner, i.e. as an optimization of the expected costs and benefits when applying the model. This is in accordance with ISO 14971, where risks are a combination of probability and severity of a potential harm [8]. 

Such approaches are linked to the field of decision theory [17]. Here, the term utility is usually applied instead of the risk-benefit relationship. This means, that an application specific utility function has to be defined which represents the relationship between benefits and costs respectively the overall utility of the decision problem. This utility function has to be optimized to achieve the best outcome. This approach can be combined with a risk analysis and its associated risk factors [18, 19]. In particular, this was applied to classification problems in medical applications [13, 20, 21] as well as to medical decision making in a general context [22]. Additionally, it was proposed as a basic rationale for optimizing ML models [23]. This approach converts the construction of the ML model into a process for finding an optimal decision rule based on probabilities and weights (i.e. costs or utilities) of the corresponding risks and benefits.

The current paper follows this approach for evaluating the performance of ML models based on risk profiles of the specific clinical application and integrating such methods into the development of ML-based medical devices. We analyze the impact, that results from variations in risk profiles. The paper focuses on binary classification tasks and subsequently on the evaluation of the outcome in terms of appropriate performance metrics. We aim at clarifying the relationship between risk management and performance assessment. This includes the application of regulatory requirements for the integration of risk considerations across the entire lifecycle of an ML-based medical device, where the development of the ML model is a crucial part. For this purpose, the paper includes the analysis of the following three research questions:

- Research Question A: First, we analyze how many recent scientific papers about using ML in medical applications only use standardized performance metrics without including the (clinical) impact of application-specific risks. Here, the term recent refers to a selected reference period of time (1 year backwards in time starting at the date of the literature research for this research). Additionally, we restricted the analysis to ML-based binary classification tasks, since this was the main focus of our paper.
- Research Question B: Second, we analyzed the differences when applying standardized performance metric, that only include accuracy / error rates, in comparison to an approach which includes the impact of different risks in the performance metric. The main question was to identify how big the differences can be when applying the different strategies, i.e. standardized vs. risk-based approach.
- Research Question C: Third, the integration of the overall results was assessed in relation to the requirements given by the corresponding standards and regulations, with a focus on the requirements in the EU. This refers to the question what current and upcoming EU regulations and standards require for the development of ML-based medical devices, in particular regarding the assessment and management of risks and their integration into the development and evaluation process. Additionally, it was analyzed how risk-based performance metrics are able to fulfill the requirements. Preliminary results for the second of these topics were presented in [24]. This included a basic model for assessing the impact of risk factors on the outcome of ML-based classification methods. The analysis was substantially extended in this new paper with respect to each of the research questions described above.

Remark – usage of important terms in this paper:

- In this paper, the term validation basically refers to the fine tuning of ML models / selection of hyperparameters, as it is commonly used in the ML community. Besides training and testing, this is considered to be a central step during the development of an ML model [7]. In order to avoid confusion, we like to emphasize that validation is used with another meaning in regulations and standards applicable to medical devices like the MDR [6]. In these classical terms, validation means “… establishing by objective evidence that device specifications conform with user needs and intended use(s)” [25]. In this sense, validation does not only refer to a tuning of models using independent data but to a proof that the technical criteria meet the needs of the particular application. The assessment of the application-specific outcome, in particular including risks and benefits, is a crucial part of this step [6].
- According to the corresponding regulations and standards like MDR and ISO 14971, the development of an ML model is an integral part of the product life cycle of the corresponding medical device [6, 8]. Thus, the regulatory requirements already apply to these phases. In research papers, the analysis and application of regulatory requirements, often seems to be considered as a separate step which can be performed later. In particular, this applies to risk management or validation (in the more general sense of the regulations for medical devices). However, this is not actually in compliance with the regulations, when a model is used in a medical device. For example, risk management and validation (again in the more general sense) need to be addressed according to a dedicated plan (i.e. risk management and validation plan). This plan needs to be set up in the initial phases of the development process [8]. Within our paper, we want to emphasize this approach. In this direction, validation means that an assessment of an ML model, that takes clinical aspects into account, has to be integrated in the entire development process. This should not be a post-hoc development step, but an integral part of the entire process.

## Methods

The following sections describe the basic methodology as it was applied in this paper for each of the three research questions. The results are presented later in the corresponding sections of Results.

### Research question A – utilization of risk-based performance metrics in recent scientific publications

As a first step, we hypothesize that most scientific publications about machine learning techniques only apply standardized metrics and do not include use-case specific costs, benefits, or risk factors into their assessment of model performance. According to the definition of the research question, the analysis was restricted to binary classification tasks, since this was the main focus of this paper. We did not use a comprehensive literature research for this purpose. Such an approach would be nearly impracticable because of its wide scope. Instead, we utilized an exemplary research by collecting and analyzing a consecutive sample of eligible publications for a reference time frame. The literature research was performed in PubMed^®^ (https://pubmed.ncbi.nlm.nih.gov/) as a reference database. In order to reflect recent publications, the time period for the search was set to 1 year backwards in time starting at the date of the literature research for this research, i.e. Nov 15, 2022. The analysis was restricted to concrete use cases and studies in the field of medical applications, where binary classification was a main focus of the publication. The following search term was used: “machine learning” classification (performance OR evalua* OR assess*) metric*, where the search terms could appear in any fields. The first two parts were included to select ML-based classification tasks. The remaining part narrowed the search to cases where an assessment based on performance metrics was performed. Filters for *free full text* and *in the last 1 year*, i.e. the previous year starting from the date of the search, were added to restrict the search to the most recent and freely accessible publications. This was not considered as a major restriction since it still represents a valid cross-sectional sample of articles. Based on the *free full text* criterion, only papers were included which were freely available, at the time of the search, i.e. Nov 15, 2022. Finally, only papers in *English* were selected using another PubMed^®^ filter option.

**Fig. 1:**
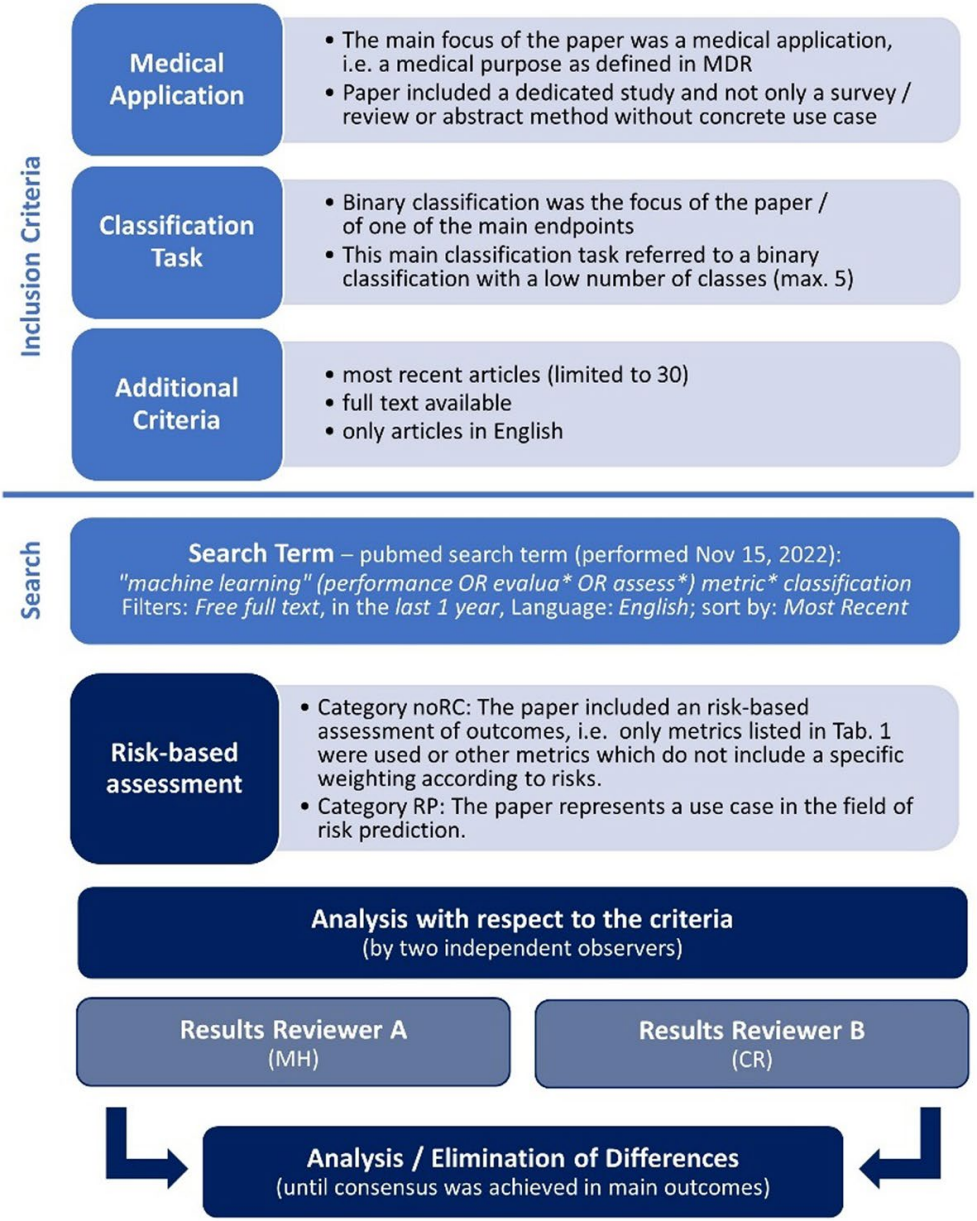
Literature review process. Method for the exemplary literature research including criteria for inclusion as well as analysis, search term, and analysis steps (until consensus was reached)

The identified articles were analyzed starting from the most recent towards the more antecedent publications until a number of 30 papers was included into the analysis. The following exclusion criteria were used to only focus on relevant publications. The evaluation of the criteria was performed by two observers (MH and CR), independently. Differences in the results were analyzed and discussed until consensus was reached. For this purpose, the relevant papers were examined together in order to sort out remaining differences in the interpretation. This process and the inclusion criteria are shown in Fig. 1. The shown categories for the risk-based assessment (i.e. noRC and RP) are described further below. The literature search was performed on Nov 15, 2022. According to the option “*in the last 1 year*”, it included papers from Nov 2021 to Nov 2022.

#### Exclusion criteria for literature research

- The main focus / task of the paper was not a direct medical application and/or did not focus on a dedicated clinical study / use case. Based on this, publications from other domains, surveys / systematic reviews, abstract presentation of methods without use case, etc. were excluded.
- Binary classification was not the focus of one of the main endpoints in the study. For borderline cases, where binary classification results were reported within a multiclass classification task, we restricted our search results to cases, where only a limited number of classes (up to 5) were addressed and the performance of the single classes was a main outcome. The rationale behind this selection was that for multiclass problems with many classes the assessment of risks is even more remote. We wanted to focus on applications where the inclusion of risk factors would be more obvious.
- The used performance metrics were listed in the paper and described in a way, that they can be judged appropriately.

Based on these criteria, the literature search provided a random sample / cross section of recent publications in this field which was further analyzed regarding the used performance metrics for the binary classification task. The literature search aimed at analyzing how many of the publications contained risk-based considerations for the evaluation of the models, in this particular sample of articles. For this purpose, a performance metric is specified to not include risk-based considerations, when it fulfills the following condition: It only contains error rates without a specific weighting or with a generic weighting, which is not adapted to risks or other clinical aspects of the specific use case. In particular, this included the following metrics, which are based on the numbers of true positives ($\:TP$), false positives ($\:FP$), true negatives ($\:TN$), and false negatives ($\:FN$) in the results of the binary classification task. Basically, the metrics listed in Table 1 were documented within our study. They were collected from [11]. Only those performance metrics were included which do not include risk-based considerations according to the provided definition.

**Table 1:** Table of standard performance metrics. This list included in [11] describes performance metrics typically used for ML-based classification tasks. Only those metrics are included, which contain no risk-based considerations according to the specification in our paper. It is assumed that the of true positives (*TP*), false positives (*FP*), true negatives (*TN*), and false negatives (*FN*) are given. See [11] for more details about the definition and utilization of these metrics

**General / overarching definitions**	
**Number of actual positive cases:** $\:{P}={T}{P}+{F}{N}$	**Number of actual negative cases:** $\:N=TN+FP$
**Number of predicted positive cases:** $\:{P}{P}={T}{P}+{F}{P}$	**Number of predicted negative cases:** $\:PN=TN+FN$
**Total Population:** $\:{P}{o}{p}={P}+{N}$	**Prevalence:** $\:Prev=\frac{P}{P+N}=\frac{P}{Pop}$
**Metrics documented in the literature research within this study**	
**Sensitivity / Recall / True Positive Rate:** $\:{T}{P}{R}=\frac{{T}{P}}{{P}}$	**Specificity / True Negative Rate:** $\:TPN=\frac{TN}{N}$
**Accuracy:** $\:{A}{c}{c}=\frac{{T}{P}+{T}{N}}{{T}{P}+{F}{P}+{T}{N}+{F}{N}}$ **or equivalently Error rate:** $\:{E}{r}{r}=1-{A}{c}{c}$	**Balanced Accuracy,** i.e. accuracy after balancing of positive / negative test samples / class members: $\:BA=\:\frac{TPR+TNR}{2}$
**Precision / Positive Predicted Value:** $\:{P}{P}{V}=\frac{{T}{P}}{{P}{P}}$	**Negative Predictive Value:** $\:NPV=\frac{TN}{PN}$
$\varvec{F_1}$**-Score:** $\:{F}1=2\cdot\:\frac{{P}{P}{V}\cdot\:{T}{P}{R}}{{P}{P}{V}+\:{T}{P}{R}}$	**other** $\varvec{F_{\beta\:}}$**-Scores:** $\:F\beta\:=\left(1+{\beta\:}^{2}\right)\cdot\:\frac{PPV\cdot\:TPR}{{\beta\:}^{2}\cdot\:PPV+\:TPR}$
**Matthews Correlation Coefficient:** $\quad\quad MCC=\sqrt{TPR\cdot\:TNR\cdot\:PPV\cdot\:NPV}-\sqrt{\left(1-TPR\right)\cdot\:\left(1-TNR\right)\cdot\:\left(1-PPV\right)\cdot\:\left(1-NPV\right)}$	**Geometric Mean:** $\:MCC=\:\sqrt{TPR\cdot\:TNR}$
**Measures which include not single models (fixed threshold) but multiple variations of thresholds**	
**Receiver Operating Characteristics** **(*ROC*) Curve,** i.e. plot of $\:{F}{P}{R}$ (on $\:{x}$ axis) vs. $\:{T}{P}{R}$ (on *y *axis).	**Precision-Recall Curve (*PRC*),** i.e. plot of recall / $\:TPR$ (on $\:x$ axis) vs. precision / $\:PPV$ (on $\:y$ axis).
**Area under the** ***ROC*** **Curve:** $\:AUROC=\int_0^1{ROC\left(x\right)\:dx\:}$ as the integral over the function $\:{R}{O}{C}\left({x}\right)$ described by the $\:{R}{O}{C}$ Curve	**Area under the** ***PRC*** **Curve:** $\:AUPRC=\int_0^1PRC\left(x\right)\:dx$ as the integral over the function $\:PRC\left(x\right)$ described by the $\:PRC$ Curve
**Measures for comparison of two predictions**	
**(Cohen’s) Kappa:** $\:{\kappa\:}=\frac{{{p}}_{0}-{{p}}_{{c}}}{1-{{p}}_{{c}}}$ where $\:{{p}}_{0}$ is the agreement between the predictions and $\:{{p}}_{{c}}$ is the agreement with respect to a random prediction	**(Cohen’s) Weighted Kappa:** (Cohens’s) Kappa $\:\kappa\:$ with additional weights included, e.g. according to risks or costs

In Table 1, only the $\:{F}_{\beta\:}$ score and the weighted (Cohen’s) Kappa allow the integration of additional weights. For the $\:{F}_{\beta\:}$ score, the factor $\:\beta\:$ determines the relation of weights between precision and sensitivity (recall). For the weighted (Cohen’s) Kappa, the weights can be more directly utilized to integrate risk factors [26]. In these cases, it has to be checked separately, whether the weighting is performed in a generic way or in a dedicated way which includes risk factors. All other metrics only depend on the $\:TP$, $\:FP$, $\:TN$, and $\:FN$ values, directly or indirectly. Within the literature study, all of these metrics (and diagrams) were collected and documented, independent of whether they had been applied in the training, validation, and/or testing phase.

For the rating of each paper, we used a subcategorization since some of the papers represented use cases where risk prediction was the basic task. In these cases, the basic goal of the ML model / system was to predict risk of medical outcomes, e.g. regarding mortality or susceptibility for certain diseases. Thus, we used a specific category for these cases. Also in this category, the assessment of the ML models can be based on standardized metrics without considering the impact of potential errors in the risk prediction. Otherwise, it can include such considerations. Finally, we arrived at the following categorization.

- noRC: no risk considerations, i.e. only standardized metrics as listed in Table 1 or other assessment methods which do not include specific weightings for potential risks.
- RC: risk considerations included, i.e. opposite of class noRC.
- RP: papers representing a use case in the field of risk prediction.
- noRP: opposite of RP.

The overall rate of publications, which included risk consideration according to our specification, was addressed as the primary endpoint. More precisely, this refers to the following percentages:

- C1) Percentage of cases which include risk predictions or other risk considerations in the assessment of outcomes, i.e. papers in categories RC or RP compared to all papers.
- C2) Percentage of cases which include risk considerations in the assessment of outcomes, i.e. papers in categories RC compared to all papers.
- C3) Percentage of papers with risk considerations within the cases, which do not present risk prediction use cases, i.e. category RC / noRP vs. all cases in noRP.

No formal hypothesis testing and a-priori estimation of statistical power was included, since we did not define a dedicated upper limit for the rate. But, an a-posteriori estimation (one-sided 95% confidence interval) for the inclusion of risk factors was performed assuming a binomial distribution. The one-sided interval was used since we were basically interested to determine an upper limit for publications including risk-based considerations. For this purpose, the binom.test function from the R statistical computing package (version 4.0.5, The R Foundation for Statistical Computing, Vienna/Austria) was applied using the Clopper-Pearson option for calculating the confidence interval.

### Research question B – impact of risk factors into performance metrics

As a second research question, the impact of risk factors was assessed, when they are integrated into performance measures for binary classification tasks. For this purpose, an artificially constructed model was utilized for the error distributions, in this paper. More specifically, the artificial model, which is based on modified Gaussian functions, represents false positive and false negative rates depending on a threshold parameter in an ML-based classification model. This means that we do not train a concrete ML model. Instead, we assume that the ML model results in the error rates represented by the artificial model. Details about this model and its subsequent usage in accuracy metrics are presented further below. These resulting accuracy measures include dedicated weight factors which represent the costs of the different types of errors. This reflects a limited version of the full decision theoretic approach as proposed in [17, 22]. Instead, it was more directly adjusted towards its use in ML-based classification tasks. In particular, the model was coupled to the corresponding $\:ROC$ curves, for this purpose. In comparison to references like [17, 22, 23], we utilized a different notation which does not require the full background about decision theory and utility functions, but provides a self-explanatory description.

In this paper, the following artificially constructed model was used for representing the error distributions and subsequently the particular performance metrics. The model includes a parameter which can be utilized to vary the results, in a systematic way. This allows to measure the impact of different error characteristics. A generic setup was used where a classifier $\:F$ is predicting the binary outcome $\:Y\in\:\left\{\text{0,1}\right\}$ from a set of input features $\:X$, i.e. the prediction is performed according to $\:\widehat{Y}=F\left(X\right)$. This prediction was applied to a set of data $\:\left({X}_{i},{Y}_{i}\right)$, where the $\:{Y}_{i}$ were considered as the ground truth, i.e. the correct classification values for the input values $\:{X}_{i}$. The $\:\left({X}_{i},{Y}_{i}\right)$ could represent training, validation, or testing data. We do not refer to a concrete classifier, here. We just analyze potential outcomes of a generic kind of classifier, which is not actually implemented. Additionally, it was regarded that the classifier depends on a threshold $\:s$. Thus, a particular instance of the classifier can be represented by a binary-valued function $\:F\left(s,X\right)$ which includes the threshold $\:s$ as a parameter. As already mentioned, we utilized an artificially constructed error distribution to demonstrate the behavior of performance metrics when the adjustable parameters of the artificially constructed error distributions get changed. This means, that we assumed that the false positive $\:FPR\left(s\right)$ and false negative rates $\:FNR\left(s\right)$ are given by a parametric function. We used modified Gaussian functions of the following form, for this purpose.

1 $$\:FPR\left(s\right)=\left(1-s\right)\cdot\:\text{e}\text{x}\text{p}\left(-\frac{{s}^{2}}{{\sigma\:}_{FP}}\right)$$

2 $$\:FNR\left(s\right)=s\cdot\:\text{e}\text{x}\text{p}\left(-\frac{{\left(1-s\right)}^{2}}{{\sigma\:}_{FN}}\right)$$

**Fig. 2:**
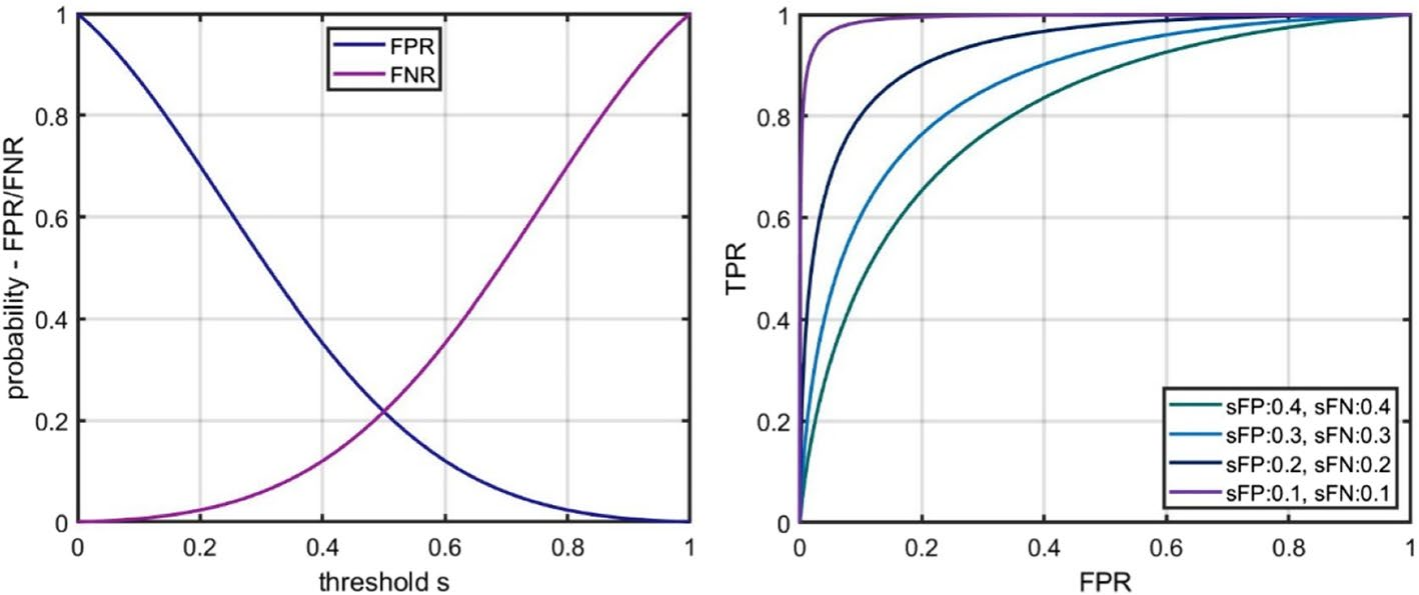
Artificial model of error distributions – graphical representations. Left side: Artificial model of error distributions, i.e. $\:FPR\left(s\right)$ and $\:FNR\left(s\right)$ in dependence of the threshold *s.* The model is based on the modified Gaussian functions as defined in Equations (1) and (2), i.e. of the form $\:FPR\left(s\right)=\left(1-s\right)\cdot\:\text{e}\text{x}\text{p}\left(\frac{{s}^{2}}{{\sigma\:}_{FP}}\right)$ and $\:FNR\left(s\right)=s\cdot\:\text{e}\text{x}\text{p}\left(\frac{{\left(1-s\right)}^{2}}{{\sigma\:}_{FN}}\right)$, where fixed parameters $\:{\sigma\:}_{FN}=0.3$ were used. Right side: Resulting $\:ROC$ curves for a set of different parameters, $\:{\sigma\:}_{FN}=0.1, \:{\sigma\:}_{FP}=\:{\sigma\:}_{FN}=0.2,\:{\sigma\:}_{FP}=\:{\sigma\:}_{FN}=0.3$ and $\:{\sigma\:}_{FP}=\:{\sigma\:}_{FN}=0.4$, where sFP refers to $\:{\sigma\:}_{FP}$ and sFP to$\:{\sigma\:}_{FN}$, in the legend

The included terms $$\:\left(1-s\right)$$ and $$\:s$$ modify the Gaussians in a way that $$\:FPR\left(1\right)=FNR\left(0\right)=0$$. These models were selected since the resulting ROC curves represent typical courses of ROC curves. The scaling parameters $$\:{\sigma\:}_{FP}$$ and $$\:{\sigma\:}_{FN}$$ can be used to adjust the achieved quality of the classifier. This can be seen in the corresponding ROC curves. Figure 2, left part shows the course of the error distributions along the threshold $$\:s$$ and for the parameter set $$\:{\sigma\:}_{FP}=$$$$\:{\sigma\:}_{FN}=0.3$$. On the right side, the corresponding $$\:ROC$$ curves are shown for varying parameters $$\:{\sigma\:}_{FP}$$ and $$\:{\sigma\:}_{FN}$$. Mind that the threshold $$\:s$$ is only encoded implicitly, in the $$\:ROC$$ curve representation.

As a next step, a risk model was constructed which assigns certain “costs” to the different types of errors *FP* and $\:FN$. These costs reflect the impact of the particular risks which are caused by the corresponding type of error. We assume costs $\:{w}_{FP}$ and $\:{w}_{FN}$, which are fixed weights. In the current paper, we do assume no costs for the cases of correct classifications, but only for the error cases. In terms of conditional probabilities $\:\text{P}\left(\widehat{Y}\left|Y\right.\right)$, the resulting expected risk $\:ER\left(s\right)$ can be calculated according to

3 $$\:ER\left(s\right)=E\left({w}_{FP}\cdot\:\text{P}\left(\widehat{Y}=1\left|Y=0\right.\right)+{w}_{FN}\cdot\:\text{P}\left(\widehat{Y}=0\left|Y=1\right.\right)\right),$$

where $\:E\left(\cdot\:\right)$ denotes the expected value. For given numbers of positive and negative cases, i.e. $\:P$ and $\:N$, the expected risk can be calculated as

4 $$\:ER\left(s\right)={w}_{FP}\cdot\:N\cdot\:FPR\left(s\right)+{w}_{FN}\cdot\:P\cdot\:FNR\left(s\right).\:$$

Positive and negative cases refer to the true situation, i.e. true prevalence, and not the predictions. Only these relationships reflect the actual use case. Basically, the expected risk $\:ER\left(s\right)$ can be considered as a negative version of a utility function, since it represents some kind of costs instead of utilities / benefits. This is consistent with the general definition in normative decision theory [23]. According to this approach, the expected utility $\:EU\left(s\right)$ is defined as the sum of utilities $\:U\left(r\right)$ across all potential outcomes $\:r$ from a set $\:R$ of results weighted by the respective probabilities $\:\text{P}\left(\left.\text{R}\text{e}\text{s}\text{u}\text{l}\text{t}\left(s\right)=r\right|s\right)$, i.e.

5 $$\:EU\left(s\right)=\sum\limits_{{}_{r\in\:R}}U\left(r\right)\cdot\:\text{P}\left(\left.\text{R}\text{e}\text{s}\text{u}\text{l}\text{t}\left(s\right)=r\right|s\right).$$

$\:\text{P}\left(\left.\text{R}\text{e}\text{s}\text{u}\text{l}\text{t}\left(s\right)=r\right|s\right)$ represents the probability, that the outcome $\:r$ occurs, when a given parameter or threshold $\:s$ is used. For the results set $\:R=\left\{FP,FN\right\}$, we obtain the relationships $\:U\left(FP\right)={w}_{FP}$, $\:U\left(FN\right)={w}_{FN}$, $\:\text{P}\left(\left.\text{R}\text{e}\text{s}\text{u}\text{l}\text{t}\left(s\right)=FP\right|s\right)=\:\text{P}\left(\widehat{Y}=1\left|Y=0\right.\right)=N\cdot\:FPR\left(s\right)$, and $\:\text{P}\left(\left.\text{R}\text{e}\text{s}\text{u}\text{l}\text{t}\left(s\right)=FN\right|s\right)=\:\text{P}\left(\widehat{Y}=0\left|Y=1\right.\right)=P\cdot\:FNR\left(s\right)$. This represents the basic relationship between our approach and normative decision theory. Mind that in our case, we used costs instead of utilities. This clarifies in which way the expected risk $\:ER\left(s\right)$ represents a negative version of a utility function.

For finding the best threshold $\:s$, the expression $\:EU\left(s\right)$ has to be maximized respectively $\:ER\left(s\right)$ minimized. We can apply a monotone transformation on $\:ER\left(s\right)$ without changing the relationships between $\:ER$ values and thus also the optimization procedure. In general, linear transformations do not substantially change a utility function [23]. In particular, a linear transformation of the following form can be applied to obtain modified, but equivalent values $\:\stackrel{\sim}{ER}\left(s\right)$:

6 $$\:\stackrel{\sim}{ER}\left(s\right)=\frac{1}{{w}_{FP}\cdot\:N}ER\left(s\right)=FPR\left(s\right)+\frac{{w}_{FN}\cdot\:P}{{w}_{FP}\cdot\:N}\cdot\:FNR\left(s\right).\:$$

Using the relative proportion

7 $$\:{c}_{FN}=\frac{{w}_{FN}\cdot\:P}{{w}_{FP}\cdot\:N}\:,$$

this modified version can be written in a simpler form as

8 $$\:\stackrel{\sim}{ER}\left(s\right)=FPR\left(s\right)+{c}_{FN}\cdot\:FNR\left(s\right).\:$$

Subsequently, $\:{c}_{FN}$ is called risk ratio as it reflects the relationship between the error types $\:FN$ and $\:FP$. Such a simplification, where only the relative ratio of risk values is considered, is limited to the case when only two risk factors are regarded. $\:\stackrel{\sim}{ER}\left(s\right)$ will still be called expected risk since it is equivalent to $\:ER\left(s\right)$ with regard to risk minimization as given in the following formula. In other words, the formula determines the threshold $\:s$ which optimizes the expected risk, i.e.

9 $$\:s=\underset{s}{\text{argmin}}\ ER\left(s\right)=\underset{s}{\text{argmin}}\stackrel{\sim}{ER}\left(s\right)=\:\underset{s}{\text{argmin}}\left(FPR\left(s\right)+{c}_{FN}\cdot\:FNR\left(s\right)\right).\:$$

**Fig. 3:**
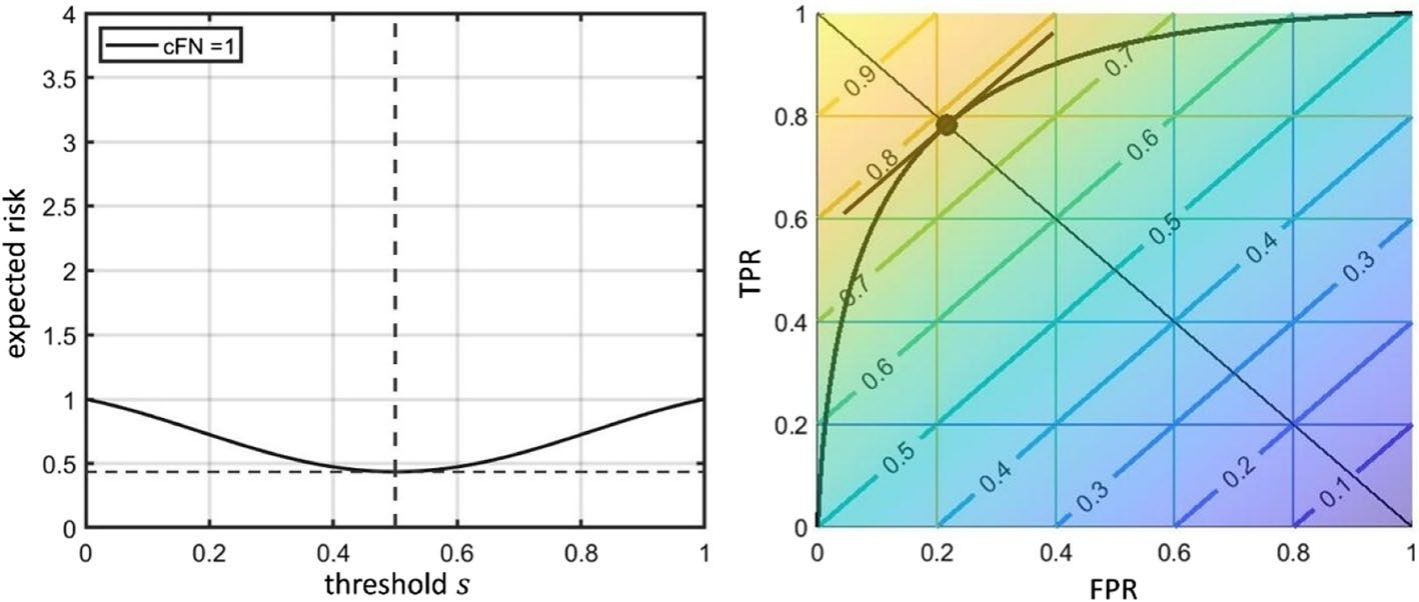
Optimization of expected risk. Left side: Representation of the threshold optimization with respect to the expected risk $\:\stackrel{\sim}{ER}$ using a diagram where the $\:x$ axis represents the threshold variable $\:s$ and the $\:y$ axis the $\:\stackrel{\sim}{ER}\left(s\right)$ function. The same artificial model was used as in Fig. 2, left side (i.e. with parameters $\:{\sigma\:}_{FP}=\:{\sigma\:}_{FN}=0.3$). The optimum threshold is the point where $\:\stackrel{\sim}{ER}\left(s\right)$ reaches its minimum. Right side: $\:\stackrel{\sim}{ER}\left(s\right)$ diagram for the same model with the weighted balanced accuracy metric ($\:WBA$, see description below) metric overlaid in a color coding. Additionally, the contour lines of the metric are displayed. The optimization of $\:WBA$ is equivalent to finding the optimum threshold for the expected risk $\:\stackrel{\sim}{ER}$. In the representation on the right side, (local) optimization is equivalent to finding the points on the $\:ROC$ curves which are tangent to the iso-contour lines of the function $\:WBA$ (depicted by the dot). This tangent is shown in brown color (parallel to the iso-contour lines). The concrete $\:\stackrel{\sim}{ER}$ values are directly shown on the iso-contour lines (colored lines, which are straight lines in this case). The diagonal line represents the symmetry line between positive and negative cases

This turns the task of finding the threshold for the binary classification problem into a decision problem with respect to the expected risk. In contrast to many standard scenarios in decision theory, it is not a decision between a set of discrete alternatives or actions but between different values of the threshold $\:s$ coming from a continuous range of alternatives. However, it remains the decision for a certain value under the uncertainties given by the particular risks. This procedure can be represented as shown on the left side of Fig. 3. Here, the expected risk $\:\stackrel{\sim}{ER}\left(s\right)$ for the artificial model given by Eqs. (1) and (2) is plotted against the threshold value. The optimum threshold is the point where the function $\:\stackrel{\sim}{ER}\left(s\right)$ achieves its minimum. The position of the minimum is shown by the dotted line. Due to the symmetry of the artificial model, this line lies at $\:s=0.5.$

The expected risk can be considered as a performance metric for classifiers which integrates a risk-based weighting to the error rates. In contrast to usual metrics, the lower values describe a better performance since errors are counted and not the rate of correct assignments. However, this can be converted into each other. For this purpose, we apply another linear transformation to obtain a metric with normalized values between 0 and 1 as shown in Equation (10) below. This metric represents a weighted version of the balanced accuracy metric $\:BA=\frac{FPR\left(s\right)\:+\:FNR\left(s\right)}{2}$. Subsequently, this metric called weighted balanced accuracy ($\:WBA$). In the WBA metric, the weight factors $\:{w}_{TP}=\frac{1}{1+{c}_{FN}}$ (for $\:TPR$) and $\:{w}_{TN}=\frac{{c}_{FN}}{1+{c}_{FN}}$ (for $\:TNR$) add up to 1, i.e. $\:{w}_{TP}+{w}_{TN}=1$.

10 $$\begin{array}{l}\:WBA\left(s\right)=\frac{1+{c}_{FN}-\stackrel{\sim}{ER}\left(s\right)}{1+{c}_{FN}}=\frac{1+{c}_{FN}-\left(FPR\left(s\right)+{c}_{FN}\cdot\:FNR\left(s\right)\right)}{1+{c}_{FN}}\\\:=\frac{\left(1-FPR\left(s\right)\right)+{c}_{FN}\cdot\:\left(1-FNR\left(s\right)\right)}{1+{c}_{FN}}=\:\frac{TPR\left(s\right)+{c}_{FN}\cdot\:TNR\left(s\right)}{1+{c}_{FN}}\\\:=\frac{1}{1+{c}_{FN}}\cdot\:TPR\left(s\right)+\frac{{c}_{FN}}{1+{c}_{FN}}\cdot\:TNR\left(s\right)={w}_{TP}\cdot\:TPR\left(s\right)+{w}_{TN}\cdot\:TNR\left(s\right).\end{array}$$

This guarantees that the maximum value of this metric equals $\:1$ as well. Due to the relationship $\:{c}_{FN}=\frac{{w}_{FN}\cdot\:P}{{w}_{FP}\cdot\:N}$, the weights are basically determined by the true prevalence as well as the relationships of the costs $\:{w}_{FN}$, $\:{w}_{FP}$ between the particular types of errors. Prevalence refers to the relationship between actual positive and the total number of cases, As long as the risk ratio $\:{c}_{FN}$ equals $\:1,$ the expected risk is equivalent to the balanced accuracy $\:BA$. $\:{c}_{FN}=1$ reflects the situations where the effects of prevalence and risk weighting balance out, i.e. when

11 $$\:{w}_{FP}\cdot\:N={w}_{FN}\cdot\:P.\:$$

This relationship will be utilized later in Research Question C – Integration into the development process for ML-based medical devices when considering standard schemes for risk assessment.

A graphical representation of this weighted balanced accuracy metric $\:WBA$ is shown on the right side of Fig. 3, in combination with the $\:ROC$ curve. $\:WBA$ is depicted using a color coding which represents the value of the function (yellow / light colors represent the highest values). Additionally, the iso-contour lines of this function are portrayed in order to make the course of the function better accessible. In this representation, optimization with respect to the threshold is the same as finding the points on the $\:ROC$ curve which are tangent to the $\:WBA$ or equivalently the $\:\stackrel{\sim}{ER}$ function. A selection of the tangent at the point with the highest $\:WBA$ (or lowest $\:\stackrel{\sim}{ER})\:$value has to be performed in the case of multiple local optima. In the diagram, the optimum point of the $\:ROC$ curve with respect to the used $\:WBA$ metric is shown as a dot. In this diagram, the symmetry is characterized by the diagonal line. Mind, that the threshold $\:s$ is not encoded explicitly here. It is only given implicitly by the correspondence between the points on the $\:ROC$ curve and the corresponding threshold values for the analyzed model.

**Fig. 4:**
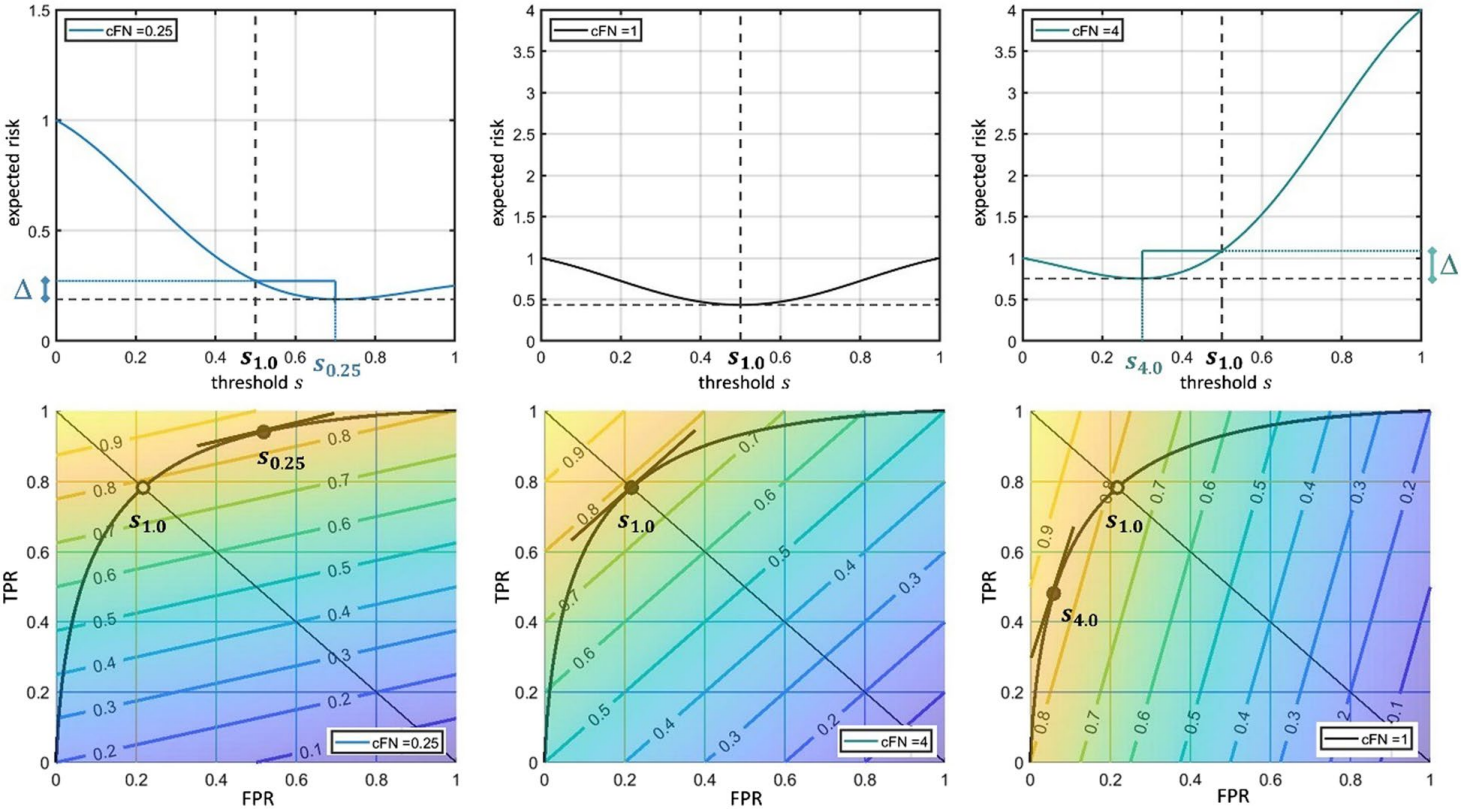
Comparison of risk ratios. Upper row: Impact of different risk ratios $c_{FN}=\;0.25,\;1.0$, and $4.0$ (from left to right) on the threshold selection and the resulting expected risk $\widetilde{ER}\;\left(s\right)$ which is shown on the y axis. The same artificial error distribution was used as in Fig. 2. The default threshold $s\;=\;0.5$ (for the case $c_{FN}=1.0$) and the corresponding expected risk is depicted as the black dashed line in all three cases. The difference between this default and the true optimal threshold $s_{0.25}$ and $s_{4.0}$ is shown by the additional blue (for $c_{FN}=0.25$) and turquoise (for $c_{FN}=4.0$) lines. The resulting difference in the $\widetilde{ER}\;\left(s\right)$ values is marked by the symbol △. Mind that a different scaling of the $y$ axis was used in the $c_{FN}\;=\;0.25$ case in order to better visualize the differences. Bottom row: curves for the same cases enriched with the (weighted balanced accuracy) metric. A color coding and the corresponding contour lines are used to visualize the course of the function. The optimum points in $ROC$ space for the particular risk ratios $c_{FN}$ (again named $s_{0.25}$ and $s_{4.0}$) are given by the black dots. They represent the points where the tangent of the $ROC$ curve and the iso-contour of the $WBA$ metric coincide. The white dot refers to the default threshold $s\;=\;0.5$ and makes the differences of the threshold estimation visible.

As a next step, the impact of different risk ratios was analyzed for the model given in Fig. 2 respectively in Eqs. (1) and (2). This serves as an example to demonstrate the analysis method. For this purpose, it was assumed that the optimum threshold $\:{s}_{1.0}$, i.e. the optimum threshold for the parameter setting $\:{c}_{FN}=1.0$, had been determined using an $\:\stackrel{\sim}{ER}$ function without a risk-based weighting, i.e. when $\:{c}_{FN}=1.0$. Basically, this leads to a metric which is equivalent to the balanced accuracy $\:BA$. Then, this threshold $\:{s}_{1.0}$ was applied to the $\:\stackrel{\sim}{ER}$ function with a risk-based weighting included, i.e. $\:{c}_{FN}\ne\:1$. In this example, $\:{c}_{FN}=0.25$ and $\:{c}_{FN}=4.0$ was used, for demonstration purposes. The resulting value $\:\stackrel{\sim}{ER}\left({s}_{1.0}\right)$ was compared to the situation where the thresholds $\:{s}_{0.25}$ and $\:{s}_{4.0}$ (i.e. the optimum threshold for the parameter setting $\:{c}_{FN}=0.25$ and $\:{c}_{FN}=4.0$) would have been used. This refers to the situation, when the expected risk would have been obtained with the correct weight $\:{c}_{FN}\ne\:1$. The effect of this variation is shown in Fig. 4. In the upper row, the $\:\stackrel{\sim}{ER}\left(s\right)$ values were plotted against the threshold $\:s$. For comparing the results, the threshold $\:{s}_{1.0}$ as well as the height of the expected risk at $\:{s}_{1.0}$ was included in the diagrams for $\:{c}_{FN}=0.25$ and $\:{c}_{FN}=4.0$. The optimum thresholds and corresponding expected risks are shown by the blue (for $\:{c}_{FN}=0.25$) and turquoise (for $\:{c}_{FN}=4.0$) line elements. The resulting difference between the risk values (at $\:{s}_{1.0}$ vs. $\:{s}_{{c}_{FN}}$) is shown by the Δ symbol at the side.

In the bottom row of Fig. 4, the situation is shown using the $\:ROC$ curves enriched with the $\:WBA$ metric. The iso-contours remained straight lines but their slope changed according to the different weights of positive and negative cases. This had an impact on the determination of the optimum points, since the tangents between the $\:ROC$ curve and the iso-contours now match at another positions. In the diagram, this is shown by the brown tangent lines. The resulting points $\:{s}_{0.25}$, $\:{s}_{1.0}$, and $\:{s}_{4.0}$ were depicted by black dots. They represent the optimum points in $\:ROC$ space with respect to the particular $\:WBA$ metric. It can be seen, that the optimum now deviates from the diagonal symmetry line. For the cases with $\:{c}_{FN}\ne\:1$, the default threshold $\:{s}_{1.0}$, i.e. the threshold for the case $\:{c}_{FN}=1$, is shown as a white dot.

This describes the basic approach for our analysis. This was applied to a more comprehensive setting in order to systematically elaborate the effect of different risk ratios on the expected risk and the associated metrics. For this purpose, the risk ratio was systematically varied from $\frac{1}{16}={2}^{(-4)}$ to $16={2}^{4}$. The increment for the risk ratios between the steps was given by a factor of $2$ to achieve a fixed grid with a logarithmic scale. Additionally, the risk ratios $\:{c}_{FN}=0.1$ and $\:{c}_{FN}=10.0$ were included, since they represent important references with respect to the application of risk management in medical devices. A ratio of $\:0.1$ respectively $\:10.0$ often refers to a substantial change in the risk profile, as it will be described later in Research Question C – Integration into the development process for ML-based medical devices. Further on, the parameters of the artificial model / error distribution, as given by the modified Gaussians in Equations (1) and (2), were varied. The parameter sets ${\sigma}_{FP} = {\sigma}_{FN}=0.1$, ${\sigma}_{FP} = {\sigma}_{FN}=0.2$, ${\sigma}_{FP} = {\sigma}_{FN}=0.3$ and ${\sigma}_{FP} = {\sigma}_{FN}=0.4$ were used. These values were selected since the resulting ROC curves represent a wide spectrum of relevant curves, as it can be seen in Fig. 2. The overall relative difference in $\:\stackrel{\sim}{ER}\left(s\right)$ values when applying these changes was the main endpoint of this part of the study. This was not related to a specific application, but in a general way, since the relationship basically depends on the risk ratio $\:{c}_{FN}$. The implementation of the calculations was performed using Matlab (version R2021a, The MathWorks Inc., Natick/ Massachusetts).

### Research question C – integration into the development process for ML-based medical devices

For research question C, we analyzed which regulatory requirements have to be fulfilled regarding the assessment of model performance of ML-based classification tasks. We focused on the interplay between model evaluation, clinical impact, and risk assessment. In particular, we asked: How do the regulatory requirements apply to the definition of metrics which should be used to assess the clinical performance of an ML-based model / medical device. The basic approach was to analyze the current and upcoming regulations as well as standards required for the development of ML-based medical devices. Further on, we applied them to specific use cases. Additionally, we analyzed how the approach described in Research Question B – Impact of risk factors into performance metrics is able to fulfill the given regulatory requirements.

For this purpose, the analysis in this paper focused on the requirements in the European Union (EU). Thus, the Medical Device Regulation (MDR) [6] was considered as the central reference. Subsequently, the corresponding (harmonized) standards have to be respected as well. For risk management, this is ISO 14971 [8]. Additionally, the technical report ISO/TR 24971 [9] was taken into account. It provides further guidance how to implement risk management into the development of medical devices. BSI/AAMI 34971 was not directly used, since it does not address the core topics which are relevant for the basic risk management process. As a second upcoming regulation, the proposed AI Act of the EU [7] and its relevant requirements, e.g. regarding risk management, data governance, or quality management, were included.

Remark: The MDR as an EU regulation represents a legally binding document. Standards like ISO 14971 and ISO/TR 24971 are considered to represent the state-of-the-art and thus provide a guide to the implementation of the corresponding legal requirements. Neither the MDR nor ISO 14971 and ISO/TR 24971 provide specific requirements for ML-based medical devices. BSI/AAMI 34971 is a new technical report for the implementation of risk management in ML-based medical devices. It provides further guidance, e.g. regarding specific risks in this field. It does not address core elements of the risk management process but refers back to ISO 14971 and ISO/TR 24971, for this purpose. The AI Act includes further requirements for risk management in AI-based products. But at the moment, no standards are available yet, which are harmonized with the AI Act. Thus, there is no solid reference for implementing the requirements of the AI Act. Only the AI Act itself delivers an adequate guidance for these horizontal requirements.

The analysis for the research question C focuses on the following particular requirements / aspects which are substantiated in the mentioned regulation and standards, i.e [6–9, 27].

- Classification of the use case / ML-based medical device according to the MDR and the upcoming AI Act.
- Basic requirements for risk management and its application to classification tasks including.
  - ◦ General principles for risk management according to the MDR.
  - ◦ Additional requirements from the AI Act.
  - ◦ Representativity of data in training, validation, and testing steps.
  - ◦ Specification of risk categories according to ISO 14971 and ISO/TR 24971.
  - ◦ Assessment of risks based and utilization of a risk matrix according to ISO 14971 and ISO/TR 24971.
  - ◦ Relation between risk-based performance metrics to the risk matrix according to ISO 14971 and ISO/TR 24971.
  - ◦ Relationship between the risk matrix and the necessity to reduce risks.
  - ◦ Requirement to differentiate between hazardous situations, hazards, and harms.
- Application of the identified regulatory requirements to the concrete use scenarios.
- Overall applicability and consistency of the developed risk-based approach in relation to the regulatory requirements in the EU.

The impact of these regulatory requirements was analyzed for the following two main applications and subordinate use scenarios. For each application, a series of modifications was included to demonstrate the impact of different risk factors on assessment of model performance.

#### Use scenarios

A. *diagnostic test*: ML-based system which is integrated into a screening test for a specific disease (e.g. a specific type of cancer). Unless otherwise stated (in the subcases), the ML-based device is considered as a stand-alone test. The actual prevalence of the disease as well as the probabilities of different types of errors / risks, i.e. $\:TP$, $\:FN$, $\:TN$, and $\:FP$, are assumed to be fixed in the following subcases.
  1. Situation with very high risk in case of false negatives ($\:FN$), when an early detection of the disease is missed. For example, this can be the case, when the disease quickly develops into a critical state where the success rate of potential treatments is very limited.
  2. Situation still with high risk in case of false negatives ($\:FN$), but with an option to better detect the disease by additional tests.
  3. Situation with reduced risk in case of false negatives ($\:FN$), because the disease develops rather slowly and has less severe impact.
  4. Situation with reduced risk in case of false negatives ($\:FN$), like in scenario A3, but additionally with high risk in the case of false positives ($\:FP$). For example, this may be the case, when a biopsy or another treatment needs to be performed in the case of positively predicted cases (i.e. $\:TP$ and $\:FP$). Such additional treatments may also cause substantial harm to the patient.
B. *quality inspection*: ML-based quality assurance system for identifying deficiencies in surgical instruments before they get delivered. It is assumed that the same ratio relationships is given as in use scenario A. This refers to the relationships between positive (instrument has a defect) and negative cases (instrument has no defect) as well as error cases (i.e. $\:TP$, $\:FN$, $\:TN$, and $\:FP$).
  1. Situation where instruments with a missed detection of a defect ($\:FN$) will be delivered directly to a hospital and may cause serious harm to a patient when applied in the treatment procedure
  2. Situation as in case B1, but this time including an additional check in the hospital which substantially lowers the probability and/or severity of the potential harm of $\:FN$ cases
  3. Situation where the quality assurance step is designed to identify defects in an early production step. The particular instrument is eliminated in this case in order to reduce further financial costs, caused by $\:FP$. In this case, it is considered that additional quality steps are included to keep the $\:FN$ rate at an appropriate level, e.g. additional visual inspections or tests, which reduce the risk of delivering defect instruments / producing harm on the patient to a low and acceptable level.

## Results

### Research question A – utilization of risk-based performance metrics in recent publications

In the literature research, 115 publications were found in total, for the given search term. Starting from the most recent publication, 55 papers were analyzed, since 25 of them had to be excluded according to the given criteria, in the analysis by the first observer (MH). In the analysis performed by the second observer (CR), there were differences in 11 cases. For 9 of the 11 cases, these differences could be easily sorted out, during the subsequent discussion with both observers included. Basically, these differences were related to different naming of metrics and inaccurate application of exclusion criteria. Two cases remained, where the exclusion criteria were not perfectly clear. The first one, i.e. [28], was finally excluded, because it only addressed a natural language processing (NLP)-based approach to identify suicidal tendencies in social media and not really a dedicated binary classification task to a medical problem. This paper was replaced by the next in the list. The second paper with differing perspectives on the exclusion criteria, i.e. [29], was finally kept after the discussion between both observers. It addressed an ML based detection of postural balance to prevent falls in elderly people. This is a borderline case regarding the classification as a medical device. Since the MDR considers systems to prevent diseases as medical device, we finally classified this a medical task and thus did not exclude the paper. In none of the 11 cases, the rating of the papers, i.e. the classification into RC / no RC and RP / noRP differed.

The excluded publications and the corresponding reasons for their exclusion are provided in table S1 (supplements). Based on this, 30 papers were finally included, as defined in the search strategy. These publications were analyzed in detail. The performance metrics, used for binary classification tasks in the particular publications are listed in Table 2. In some cases, additional metrics were included which we did not have on our initial list. They were also documented in Table 2. None of them included risk factors, in a dedicated way. 

**Table 2:** Results from literature search. Table of articles which were included in the literature research regarding recent publications about performance metrics of ML-based classification models (sorted according to the “most recent” criterion). The table documents the used performance metric as well as the rating regarding the inclusion of risk-based considerations according to the specification in Research Question A – Utilization of risk-based performance metrics in recent scientific publications

First author + ref no.	Used performance metrics	Resulting category (as described in Research Question A – Utilization of risk-based performance metrics in recent scientific publications)
Ozcan [30]	Acc, Sen, Prec Additional metrics (without direct risk integration): Determinism → was neither described nor referenced reliably	noRC / noRP
Garavand [31]	Acc, Prec, Sens, Spec, F1 Score, ROC, AUROC, AUPRC	noRC / noRP
ElSeddawy [32]	Acc, Sens, Spec, F1 Score, G-mean, ROC, AUROC, (unweighted) Kappa	noRC / noRP
Kasim [33]	Acc, Prec, NPV, Sen, Spec, AUROC, (unweighted) Kappa Additional metrics (without direct risk integration): net reclassification index (NRI)	noRC / RP In this case, the basic application (mortality prediction) was strongly related to a risk-based application itself. Thus, also the evaluation included risk factors, in some sense, even though standardized metrics were used. The effect, which were caused by errors in the ML systems itself, were not included additionally.
Farhang-Sardroodi [34]	ROC, AUROC	noRC / noRP
Wu [29]	Acc, Prec, Sen, F1-Score, ROC, AUROC	noRC / noRP
Preto [35]	Acc, Prec, Sen, F1-Score, AUROC	noRC / noRP
González-Cebrián [36]	Acc, Sen, Spec, F1-Score, MCC, AUROC	noRC / RP In this case, the basic application (mortality prediction) was strongly related to a risk-based application itself. Thus, also the evaluation included risk factors, in some sense, even though standardized metrics were used. The effect, which were caused by errors in the ML systems itself, were not included additionally.
He [37]	Acc, Prec, Sen, F1-Score, ROC, AUROC	noRC / noRP
Milara [38]	Acc, Prec, Sen, Spec, F1-Score, AUROC	noRC / noRP
Emakhu [39]	Acc, Prec, Sen, Spec, MCC, F1 score, ROC, AUROC	RC / RP In this case, the basic application (Acute coronary syndrome prediction) was related to a risk-based application itself. Additionally, there was a cost-sensitive approach included in the evaluation of the models, besides the utilization of standardized metrics.
Haq [40]	Acc, Prec, NPV, Sen, Spec, ROC, Additional metrics (without direct risk integration): Dice Similarity Coefficient (DSC), Probabilistic Random Index (PRI).	noRC / noRP
Movahed [41]	Acc, Sen, Spec, F1-Score, ROC, AUROC Additional metrics (without direct risk integration): False Discovery Rate	noRC / noRP
Templeton [42]	Acc, Prec, Sen	noRC / noRP
Zou [43]	Acc, BA, Prec, Sen, Spec, F1-Score, MCC, ROC, AUROC	noRC / noRP
Tran [44]	Acc, F1-Score, ROC, AUROC	noRC / noRP
Maskew [45]	Acc, PPV, NPV, ROC, AUROC	noRC / noRP
Mabrouk [46]	Acc, BA, Prec, Sens, F1 score	noRC / noRP
Khan [47]	Acc, Prec, Sens, F1 score	noRC / noRP
Ho [48]	Acc, Prec, Sens, F1 score	noRC / noRP
Eissa [49]	Acc, Prec, Sens, MCC, F1 Score, ROC, AUROC	noRC / noRP
Salimpour [50]	Acc, Prec, Sens, (unweighted) Kappa	noRC / noRP
Berenguer-Vidal [51]	Acc, Prec, Sen, Spec	noRC / noRP
Dritsas [52]	Acc, Prec, Sens, F1 Score, AUROC	noRC / noRP
Ahmad [53]	Acc, Prec, Sen, Spec, ROC	noRC / noRP
Goñi [54]	BA, Prec, NPV, Sens, Spec, ROC, AUROC	noRC / noRP
Dubol [55]	Acc, AUROC	noRC / noRP
Hidayat [56]	Acc, Sen, Spec, ROC, AUROC	noRC / noRP
Baskozos [57]	BA, MCC, AUPRC	noRC / noRP
Shakhovska [58]	Acc, Prec, Sens, F1 Score, AUROC	noRC / noRP

This resulted in the following overall rates for the three particular endpoints.

- Category C1: Three publications [30, 34, 37] (out of a total of 30 publications) were categorized as RP. There was no additional paper which was classified as RC. Thus, the rate for category C1 was 10% (3 out of 30) with an upper limit (95% CI) of 0.24, i.e. 24%.
- Category C2: Only the paper [37] was classified as RC and RP. This leads to an overall result of 1 in 30 cases, i.e. a 3.6% rate. Here, the 95% CI was 0.16, i.e. 16%.
- Category C3: All cases in class noRP were classified as noRC. Thus, there were 0 positive out of 27 total noRP cases, leading to a 0% rate and an upper limit of the 95% CI of 0.11, i.e. 11%.

### Research question B – impact of risk factors into performance metrics

**Fig. 5:**
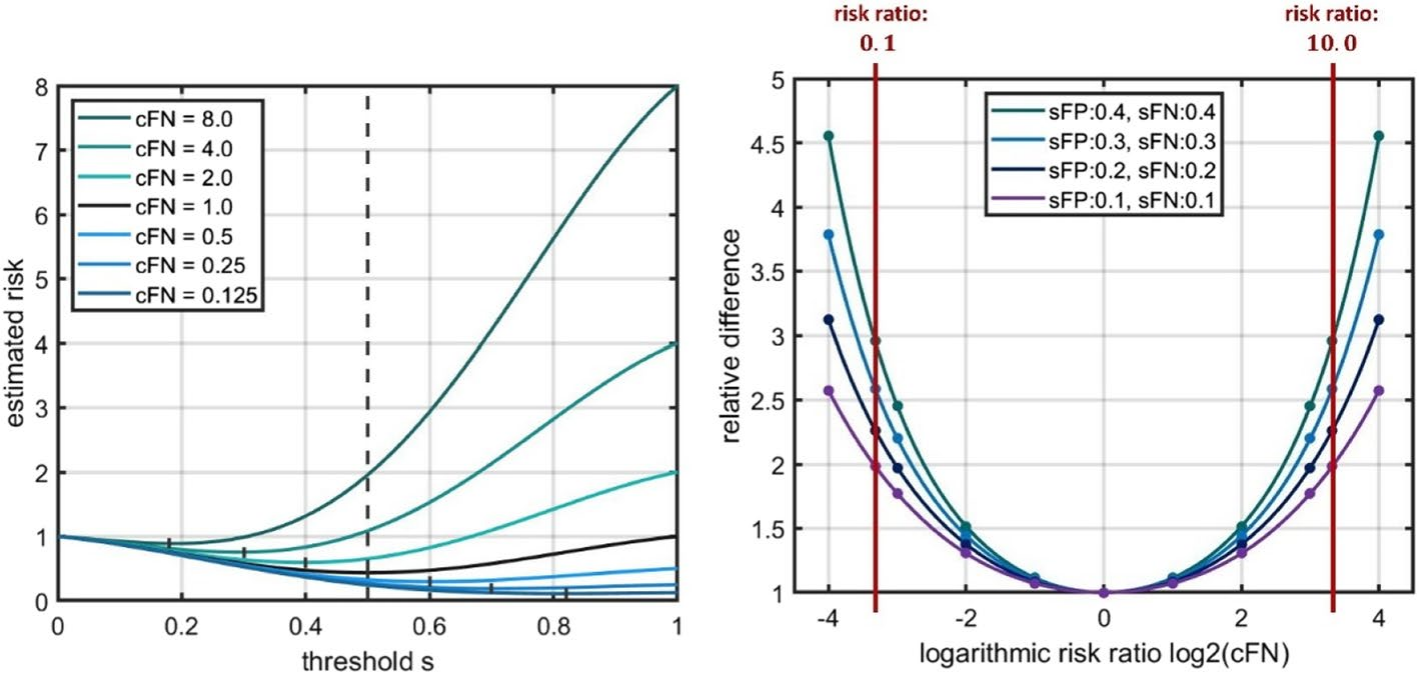
Impact of risk-based approach – graphical comparison. Graphical representation of the results given in Table 3. Left side: Visualization of the expected risk ($\:\stackrel{\sim}{ER}$) values for the particular risk ratios $\:{c}_{FN}$ in the range $\:\frac{1}{8}={2}^{-3}$ to $\:8={2}^{3}$. The same artificial model as given in Equation (1) and (2) with $\:{\sigma\:}_{FP}={\sigma\:}_{FN}=0.3$ was used

This section demonstrates how changes in the risk factors, i.e. the values for $\:{c}_{FN}$ in the $\:WBA$ metric defined in Research Question B – Impact of risk factors into performance metrics, affect the evaluation of ML classification models. More precisely, this refers to models which have error distributions represented by the artificial model for the error distributions presented in Research Question B – Impact of risk factors into performance metrics, i.e. the modified Gaussian functions. For this purpose, Table 3; Fig. 5 show the results of the expected risk $\:\stackrel{\sim}{ER}$ which were obtained. For the evaluation, the artificial model given in Equation (1) and Equation (2) was used. The expected risk values given at the default threshold $\:{s}_{1.0}=0.5$ were compared to the outcome at the optimum threshold $\:{s}_{{c}_{FN}}$ for the particular risk ratio $\:{c}_{FN}$. This means that the relative difference between $\:\:\stackrel{\sim}{ER}\left({s}_{{c}_{FN}}\right)$ and $\:\:\stackrel{\sim}{ER}\left({s}_{1.0}\right)$, i.e. the ratio $\:\frac{\stackrel{\sim}{ER}\left({s}_{1.0}\right)}{\stackrel{\sim}{ER}\left({s}_{{c}_{FN}}\right)}$ was calculated as the main outcome.

**Table 3:** Quantitative impact of risk-based approach. Differences of expected risk $\:\stackrel{\sim}{ER}$ when varying the risk ratio $\:{c}_{FN}$ systematically between $\:1.0={2}^{0}$ and $\:16={2}^{4}$ (stepwise increment by factor 2) as well as $\:{c}_{FN}=10.0$ as an extra point of evaluation. Due to symmetry reasons, the values for $\:{c}_{FN}<1.0$ are equivalent to the inverse risk ratio $\:\frac{1}{{c}_{FN}}$. The rightmost column shows the relative differences between $\:\stackrel{\sim}{ER}\left({s}_{{c}_{FN}}\right)$, i.e. the value at the optimum position $\:{s}_{{c}_{FN}}$ for the particular curve, and $\:\stackrel{\sim}{ER}\left({s}_{1.0}\right),$ i.e. the value at the default threshold $\:{s}_{1.0}$

Parameter settings of artificial model / risk ratio		Optimum threshold $\:{\varvec{s}}_{{\varvec{c}}_{\varvec{F}\varvec{N}}}$ and corresponding $\varvec{\:\stackrel{\sim}{{E}{R}}}$ value			Comparison of $\varvec{\:\stackrel{\sim}{{E}{R}}}$ values: $\:{\varvec{s}}_{{\varvec{c}}_{\varvec{F}\varvec{N}}}$ vs. default threshold $\varvec{\:{\varvec{s}}_{1.0}}$
modified Gaussian $\varvec{\:{{\sigma\:}}_{{F}{P}}\:/\:{{\sigma\:}}_{{F}{N}}}$	risk ratio / weight $\varvec{\:{c}_{FN}\:/\:{c}}$	optimum threshold $\varvec{\:{s}_{{c}_{FN}}}$	expected risk value		relative difference $\varvec{\:\frac{\stackrel{\sim}{ER}\left({s}_{1.0}\right)}{\stackrel{\sim}{ER}\left({s}_{{c}_{FN}}\right)}}$
			at $\varvec{\:{s}_{{c}_{FN}}}$: $\varvec{\:\stackrel{\sim}{ER}\left({s}_{{c}_{FN}}\right)}$	at $\varvec{\:{s}_{1.0}}$: $\varvec{\:\stackrel{\sim}{ER}\left({s}_{1.0}\right)}$	
$\:{{\sigma\:}}_{{F}{P}}=0.1,$ $\:{{\sigma\:}}_{{F}{N}}=0.1$	1.0 (default)	0.5 (default)	0.08	0.08	1.0
	2.0	0.46	0.11	0.12	1.07
	4.0	0.44	0.16	0.21	1.30
	8.0	0.40	0.21	0.37	1.77
	**10.0**	**0.38**	**0.23**	**0.45**	**1.98**
	16.0	0.36	0.27	0.70	2.58
$\:{{\sigma\:}}_{{F}{P}}=0.2,$ $\:{{\sigma\:}}_{{F}{N}}=0.2$	1.0 (default)	0.5 (default)	0.29	0.29	1.0
	2.0	0.44	0.40	0.43	1.08
	4.0	0.36	0.52	0.72	1.38
	8.0	0.3	0.65	1.29	1.97
	**10.0**	**0.26**	**0.70**	**1.58**	**2.26**
	16.0	0.22	0.78	2.44	3.12
$\:{{\sigma\:}}_{{F}{P}}=0.3,$ $\:{{\sigma\:}}_{{F}{N}}=0.3$	1.0 (default)	0.5 (default)	0.43	0.43	1.0
	2.0	0.4	0.59	0.65	1.10
	4.0	0.3	0.75	1.09	1.44
	8.0	0.18	0.89	1.96	2.20
	**10.0**	**0.16**	**0.92**	**2.39**	**2.59**
	16.0	0.08	0.98	3.69	3.78
$\:{{\sigma\:}}_{{F}{P}}=0.4,$ $\:{{\sigma\:}}_{{F}{N}}=0.4$	1.0 (default)	0.5 (default)	0.54	0.54	1.0
	2.0	0.36	0.72	0.80	1.11
	4.0	0.22	0.88	1.34	1.51
	8.0	0.08	0.98	2.41	2.45
	**10.0**	**0.04**	**1.00**	**2.94**	**2.96**
	16.0	0.00	1.00	4.55	4.55

The main results are provided in the right most column of Table 3. It can be seen that this ratio goes up to $\:2.96$, i.e. $\:196\%$ increase in expected risk, for the parameter setting $\:{\sigma\:}_{FP}={\sigma\:}_{FN}=0.4$ and the risk ratio $\:{c}_{FN}=10.0$. For $\:{c}_{FN}=16.0$, this further increases to a relative difference of $\:4.55$. The effect is lower when the risk ratio is closer to $\:{c}_{FN}=1.0$, i.e. the non-weighted case. The described effects were also reduced when the values $\:{\sigma\:}_{FP},\:{\sigma\:}_{FN}$ decreased. Such a decrease implies that the $\:ROC$ curve lies closer to an ideal classifier, i.e. a ML model which achieves $\:TPR=1$ and $\:FPR=0$, as it can be seen in Fig. 2 right side.

The results are shown graphically in Fig. 5 on the right side, using a logarithmic scaling of the $\:x$ axis, i.e. for the risk ratio $\:{c}_{FN}$. The reference values $\:{c}_{FN}=0.1$ and $\:{c}_{FN}=10.0$ are indicated by a vertical red line. Since the relative difference between$\:\:\stackrel{\sim}{ER}\left({s}_{{c}_{FN}}\right)$ and$\:\:\stackrel{\sim}{ER}\left({s}_{1.0}\right)$ is symmetric to the axis $\:{c}_{FN}=1.0$ (or equivalently $\:{\text{log}}_{2}{c}_{FN}=0$), the relative difference in expected risk is the same between a risk ratio $\:{c}_{FN}$ and its inverse $\:\frac{1}{{c}_{FN}}$. Because of this equality, the $\:{c}_{FN}$ values below 1 were omitted in Table 3. The symmetry is due to the construction of the model which has a symmetry between the positive and negative cases. On the left side of Fig. 5, the actual expected risk values $\:\stackrel{\sim}{ER}\left(s\right)$ are shown in a similar way as in Fig. 4, upper row. In this case, the different risk ratios between $\:\frac{1}{8}={2}^{-3}$ and $\:8={2}^{3}$ are integrated into one diagram. Again, the default threshold $\:{s}_{1.0}=0.5$ was marked by the dashed line. The optimum thresholds $\:{s}_{{c}_{FN}}$ for the other risk ratios are lying at the minima of the particular $\:\stackrel{\sim}{ER}$ curves. They are depicted by the vertical small dashes. Thus, the relationship between $\:\stackrel{\sim}{ER}\left({s}_{{c}_{FN}}\right)$ and$\:\:\stackrel{\sim}{ER}\left({s}_{1.0}\right)$ can be recognized with respect to the height of the particular curve at $\:{s}_{{c}_{FN}}$ (indicated by the short vertical lines at the minimum of the particular curve) and $\:{s}_{1.0}$ (i.e. the intersection between the dashed line at $\:s=0.5$ and the curve). Finally, $\:\frac{\stackrel{\sim}{ER}\left({s}_{1.0}\right)}{\stackrel{\sim}{ER}\left({s}_{{c}_{FN}}\right)}$ represents the relationship / ratio between these two height levels.

### Research question C – integration into the development process for ML-based medical devices

Based on the results of the sections before, the relation of risk-based approaches for the evaluation of ML-based medical devices in comparison to the corresponding regulatory requirements was addressed.

#### Classification of the use case / ML-based medical device according to the MDR and the upcoming AI act

According to the MDR, Scenario A describes a system with a diagnostic task as the intended medical purpose. Thus, it represents a medical device according to Art. 2 of the MDR. Additionally, it represents a software which is used to take decisions with diagnosis. This results in a classification of at least risk class IIa. Potentially, it may need to be classified as IIb or III when it may lead to a serious or also irreversible detoriation of health. These categorizations are given by classification rule 11 of the MDR. Subsequently, a third-party, e.g. notified body, needs to be included into the conformity assessment, according to the MDR [6]. This characteristic is one of the guiding principle for the classification of high-risk AI systems in the AI Act [7]. According to Art. 6 in combination with Annex II of [7], the diagnostic test in use scenario A inherits the high risk-classification, based on this rationale.

A similar classification applies to use scenario B (*quality inspection*) of Research Question C – Integration into the development process for ML-based medical devices. In this case, the ML-based system is not directly included in a medical device, but represents a part of its production system. According to the AI Act [7], the system is still considered a high-risk AI system as long as it represents a safety critical component of a medical device, which itself would be rated high-risk. A quality check for identifying deficiencies in surgical instruments, which can lead to serious harm, usually needs to be considered as a safety critical component. Additionally, the ISO 13485 [27] as the standard for quality management systems requires that tools used in the production system need to undergo a computer system validation (CSV), if they potentially lead to risks in the application of the medical device. Thus, the evaluation of the ML-based models in the use scenario A and B need to be addressed in a similar way.

#### General principles for risk management according to the MDR

As a central requirement, the evaluation of medical devices and their components has to be related to clinical performance. This is a key aspect for the development of medical devices as required in the corresponding regulations, in particular in the MDR [6]. Risks to the health of the patient have to be considered, since they constitute important clinical effects. This applies to both use cases, since both of them include potential serious harm. According to [6], the risks have to be reduced as much as reasonably possible (ALARP principle). This has to be performed unless no further substantial improvement of the risk-benefit relation can be achieved [6]. This applies to single risks as well as the overall risk of the device.

According to the MDR and ISO 14971, risk management has to be applied during the entire development process. Literally, the MDR states that “risk management shall be understood as a continuous iterative process throughout the entire lifecycle of a device.” [6] Additionally, it considers safe design as one of the first options to eliminate or reduce risks as far as possible [6]. The development of an ML-based model is a central part of the development process. Thus, risk management has to be considered, in this phase as well. According to the ALARP principle, risk reduction has to be applied within the development of the model, if substantial changes can be achieved and no other measures can keep the particular and overall risks in an acceptable range. This implies that it should be considered to include adjustments with respect to risk-based factors also in the training, validation, and testing of ML-based models, if substantial risk reduction can be achieved this way. Otherwise, the reduction of risks remains limited.

Finally, a positive risk-benefit relationship has to be guaranteed. This potentially requires to include the positive impact of properly treated cases as well. In particular, this is elaborated in ISO/TR 24971 [9]. The direct integration of benefits in the performance metrics was omitted in the present paper, as we only focused on the risk factors. However, this can easily be integrated when considering benefits as negative versions of risk factors following the approach presented in ISO/TR 24971. According to the MDR as well as ISO 14971, the evaluation should reflect the concrete use case as given in the intended use of the medical device. Risk management needs to be performed in order to mitigate risk factors in exactly this direction, where the associated application context and user / patient population as well as normal use conditions, including foreseeable misuse, have to be regarded [8]. These requirements apply for medical devices, in general. For this reason, they are also applicable to the presented use scenarios. We elaborate more details about their implementation with respect to a risk-based assessment of the corresponding ML-based models further below.

#### Additional requirements from the AI act

Within the development phase, state-of-the-art techniques in the particular domain have to be applied. For ML-based devices, this means that training, validation, and testing of the models has to be implemented according to appropriate and established performance metrics. This is reflected in the proposed AI Act of the EU [7], e.g. in its articles about risk management (Art. 9), data governance (Art. 10), and quality management (Art. 17). In Art. 9 (risk management), it is mentioned that “The testing of high-risk AI systems shall be performed, as appropriate, at any time throughout the development process, and, in any event, prior to their being placed on the market or put into service. Testing shall be carried out against prior defined metrics and probabilistic thresholds that are appropriate to the intended purpose of the high-risk AI system” [7]. Additionally, “Data sets shall take into account, to the extent required by the intended purpose, the characteristics or elements that are particular to the specific geographical, contextual, behavioral or functional setting within which the high-risk AI system is intended to be used.” (Art. 10 in [7]). Thus, it is important to consider the actual prevalence of the use case within the development and evaluation of an ML-based medical device. In Art. 10 and throughout most parts of the AI Act, the term “data sets” refers to training, validation, and test data sets.

#### Representativity of data in training, validation, and testing steps

Thus, the intended population should be addressed properly in the training, validation, and testing steps, when considering ML-based technologies. In the case of a classification task, e.g. for a disease or other deficiency, the intended population basically reflects the actual prevalence. This refers to the relative amount of positive case numbers. Thus, this number should be taken into account as a basic reference when developing an ML-based medical device. Currently, a balanced situation between positive and negative cases is often pursued for training, testing, and validation [12]. This makes sense in order to balance the unreliability in the different groups and to address the requirement for fairness / non-discrimination as e.g. included in [7]. In particular, this is important when the prevalence is a low number, e.g. the number of positive cases lies in the order of $\:{10}^{-3}$ or lower. Such a situation is given in many situations. Usually, there are much more negatives than positives in the population, since the appearance of a disease or other deficiency often is limited unless an epidemic situation occurs. The reliability of ML-based models would be rather poor, if this ratio would be represented in the corresponding data sets. Thus, it makes sense to balance them by using a higher rate of positive cases than actually given. However, the final evaluation should reflect the actual prevalence according to the requirements described above.

**Fig. 6:**
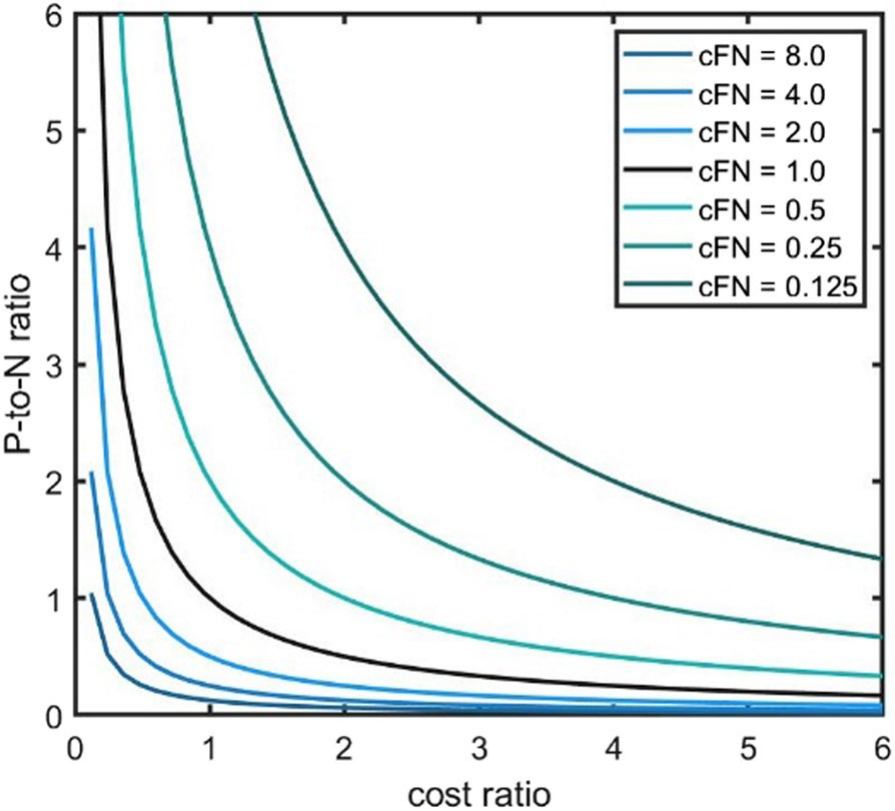
Relationship between risk ratio and P-to-N distribution. Reciprocal relationship for the overall risk ratios $\:{c}_{FN}$ (ranging from 0.125 and 8.0 with stepwise increment by factor 2). The product between the cost ratio $\:\frac{{w}_{FN}}{{w}_{FP}}$ for the particular risk and the relationship in numbers / probabilities needs to be constant to keep the overall risk at the same level

For achieving this balance, the impact / costs of different types of errors need to be considered as well. With respect to risk management, the costs are related to the severity of the (potential) harm. This has to be multiplied with the probabilities to achieve an overall estimation of risks. In a certain sense, this is reflected by Equation (7), i.e. $\:{c}_{FN}=\frac{{w}_{FN}\cdot\:P}{{w}_{FP}\cdot\:N}$, which characterizes the risk ratio as a combination of a ratio $\:\frac{{w}_{FN}}{{w}_{FP}}$ representing the costs and the ratio between negative and positive cases. The latter refers to the actual prevalence. A balanced situation occurs when the different effects are balanced out as given in Equation (11), i.e. when $\:{w}_{FP}\cdot\:N={w}_{FN}\cdot\:P.$ This means that the relation between negative and positive cases respectively $\:FP$ and $\:FN$ needs to be reciprocal to the cost ratio to keep the overall risk ratio at a constant level. This relationship is shown graphically in Fig. 6 for different overall risk ratios $\:{c}_{FN}$ between $\:0.125$ and $\:8.0$ with stepwise increment by factor 2.

#### Specification of risk categories according to ISO 14971 and ISO/TR 24971

The definition of risk as a combination of severity and probability is a central point in the risk management standard [8] and the associated guidance [9]. In general, risk is considered as a situation that may lead to a harmful effect on humans in some way. In particular, this refers to physical harm. Risk is represented by a probability (likelihood) that this harm occurs and a severity which rates the level of impact. These two aspects have to be included during risk assessment. The associated rating of risk has to reflect the final impact of the medical device, when applied in its use context. Ideally, this would be given in quantitative terms, i.e. concrete numbers for the probabilities and severities. However, it is recognized that this is often not possible in such a consequent way. Instead, it is allowed to perform risk analysis in a semi-quantitative or also qualitative way [8, 9]. The semi-quantitative approach means that the probabilities and severities of risks are grouped together in certain levels, according to a rating performed by subject experts. According to ISO/TR 24971, this should include technical, clinical, and regulatory experts as well as other relevant expertise [9]. In particular, this includes expertise in the field of quality assurance, service engineers, or post production assessment. Usually, the rating of the severities is done without giving concrete numbers. This means that severity basically is defined in a purely qualitative fashion [8, 9]. The concrete manifestation of the risk levels has to be specified and justified in the risk management plan. This plan has to be set up in the early development phases. It needs to reflect the risk profile of the considered use case, e.g. use scenario A and B. The basic methodology, i.e. the described semi-quantitative categorization, can be used as a generic approach [8, 9]. A typical example is the classification shown in Table 4 (see [9]).

**Table 4:** Example of risk categories. Semi-quantitative (with respect to probability levels) respectively qualitative (with respect to severities) classification of risks in medical devices as proposed in [9]

Levels regarding probability (likelihood) of risks	Levels regarding severity of risks
frequent:$\:\ge\:{10}^{-3}$	negligible
$\:\text{probable}:\:<{10}^{-3}\:\text{and}\:\ge\:{10}^{-4}$	minor
$\:\text{occasional}:\:<{10}^{-4}\:\text{and}\:\ge\:{10}^{-5}$	serious / major
$\:\text{remote}:\:<{10}^{-5}\:\text{and}\:\ge\:{10}^{-6}$	critical
improbable:$\:<{10}^{-3}$	catastrophic / fatal

These categories are reflected by the components in the $\:WBA$ metric. The probabilities which occur due to certain types of errors, e.g. as given by the $\:FPR$ and $\:FNR$ values, describe the probabilities (likelihoods) that a risk occurs. The particular ‘costs’ of errors reflect the severities. The risk scores $\:{w}_{FP}$ and $\:{w}_{FN}$ represent the combination of both components, i.e. probability and severity. Usually, the probability levels are given with an exponential increase between these levels, e.g. in exponential steps with respect to the power $\:10$, i.e. in levels of type $\:{10}^{-x}$. The definition in Table 4 uses such an approach.

#### Assessment of risks based and utilization of a risk matrix according to ISO 14971 and ISO/TR 24971

According to ISO/TR 24971, the relevant risks for a medical device should be collected in a risk matrix as shown in Table 5. In this matrix, the particular risks are arranged in each combination of probability and severity levels. There typically are the following three areas contained in this matrix, which represent different requirements for further treatment of risks [9].

- a red/orange area, where risks are considered as inacceptable and mandatorily need to be reduced before the medical device can be placed on the market – e.g. $\:{R}_{6}$ in Table 5
- a green area, where the risks can be regarded as insignificant and no further reduction needs to be considered – e.g. $\:{R}_{1}$, $\:{R}_{3}$, $\:{R}_{4}$ in Table 5
- a yellow area, sometimes called ALARP region, where risks need further investigation – e.g. $\:{R}_{2}$, $\:{R}_{5}$ in Table 5

The concrete ranges for the areas have to be prespecified in a risk policy, i.e. in the initial phase of the development within the risk management plan for the device [8, 9]. Thus, acceptability of risks has to be assessed according to a strategy which is defined in advance. 

**Table 5:**
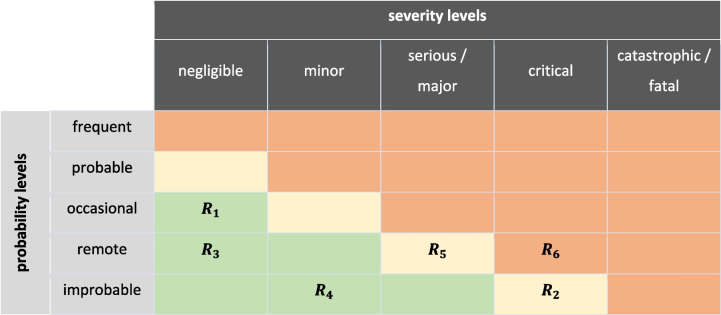
Example of risk matrices. Risk matrix based on the semi-quantitative / qualitative classification as given in Table 4. The risk matrix collects all particular risks of a medical device ( *R*_1_−*R*_6_ in this case) according to its categorization with respect to their probability and severity (basic scheme as presented in [9]). The tree different areas (red/orange – inacceptable risks, green – acceptable risks, and yellow – region where risks need further investigation) indicate which further risk management steps have to be considered before the medical device can be placed on the market

As already mentioned, the risks need to be considered as a combination between probabilities and severities. One standard approach is to calculate them by a multiplication between these two factors [59]. Other combinations may also be possible since [8, 9] do not specify further details about the combination. However, the multiplicative approach is consistent with the probabilistic method provided in Research Question B – Impact of risk factors into performance metrics as well as the normative version of decision theory. This approach is subsequently used to demonstrate the impact of different risk factors. In order to get a constant overall risk ratio, the probabilities need to be balanced with the associated severity level. This means that their product needs to be equal to a constant. In the multiplicative approach, this refers to the relationship $\:{p}_{1}\cdot\:{c}_{1}={p}_{2}\cdot\:{c}_{2}=const$ for probabilities $\:{p}_{1}, {p}_{2}$ and severities/costs $\:{c}_{1},\:{c}_{2}$. For example, this can be applied to a situation where balanced data sets are used in combination with a standard performance metric, i.e. without additional weighting. In this case, a complete balancing between cost and probability ratios is implicitly assumed. This means that the product between the severity and the probability ratio for the different types of errors is implicitly considered to equal 1.

#### Relation between risk-based performance metrics to the risk matrix according to ISO 14971 and ISO/TR 24971

The contributions of the different risk factors, e.g. $\:{R}_{1}$ – $\:{R}_{6}$ in Table 5, are usually considered to be independent items. Otherwise, the approach to reduce the risks separately would not work. Based on this, the contributions are considered to be additive. This means that the overall risk is a sum of the particular combined risks. This is in accordance with the formulas for expected risk presented in Research Question B – Impact of risk factors into performance metrics. For example, the risks, i.e. the products of probabilities and severities / costs, can be summed up into a single weight, when one risk, e.g. one type of error, shows up with multiple severity and probability levels. The same applies to a situation, where multiple aspects need to be integrated into one particular type of risk. Thus, these situations are covered by the given approach. In general, there may be a more complex combination of several effects which go beyond the scope of this paper. Within this paper, we focused on only two particular risks, namely the risk for $\:FN$ as well as the risk for $\:FP$. In this case, only the ratio $\:{c}_{FN}=\frac{{w}_{FN}\cdot\:P}{{w}_{FP}\cdot\:N}$ between them is relevant, when considering an ML-based classification task. Here, the values $\:{w}_{FN}\cdot\:P$ and $\:{w}_{FP}\cdot\:N$ aggregate the risks, i.e. severity times probability, for the particular type of error.

#### Relationship between the risk matrix and the necessity to reduce risks

The MDR requires that risks have to be reduced as far as possible without adversely affecting the benefit-risk ratio [6]. Thus, the question is when the risk level needs to be considered as decreasing substantially, according to this definition. In the case of substantially different risk categories, risk reduction would have to be applied. Additionally, it is important to clarify how this relates to the risk matrix and the risk-based assessment of medical devices. Typically, the elements at the diagonal of the risk matrix represent approximately constant levels of risk. If the probability levels are represented by an exponential scale with base $\:10$, the severity levels also need to provide such increments in order to achieve this. Thus, we assume that the difference between the severity levels is also represented by a factor of $\:10$. In summary, this difference appears between any step up in the risk matrix, either in the horizontal or in the vertical direction, i.e. when jumping from one diagonal to the neighboring one. In general, the overall risk is dominated by the risks appearing at the highest diagonal, according to the exponential scaling. The next levels constitute combined risks which are decreased by a factor of $\:10$, $\:100$, $\:1000$, etc.

#### Requirement to differentiate between hazardous situations, hazards, and harms

An additional requirement in the risk management standards ISO 14971 [8, 9] is the discrimination between hazardous situations, hazards, and harms. Harms are actual damages to humans, goods or the environment. Hazards are situations where harms may eventually occur. Hazardous situation describes a situation where humans, goods or the environment are exposed to a hazard [8]. The basic question in this section is how the differentiation between hazardous situations, hazards, and harms is related to the risk-based assessment developed in our paper.

The pure occurrence of a $\:FP$ or $\:FN$ case is not really a risk but a hazardous situation, since an $\:FP$ or $\:FN$ does not create a harm directly. For example, an $\:FN$ in an ML-based test for cancer screening indicates that a harm may result. But, it does not indicate that some level of harm actually has occurred. This may depend on the individual development of the potential disease, i.e. whether a critical or a lower stage of disease is obtained. Thus, two different factors constitute the probability of harm. For example, $\:{p}_{1}$ may represent the probability of the hazard, e.g. a $\:FP$ or $\:FN$ case, and $\:{p}_{2}$ the probability that a harm occurs when the hazard is given. The overall probability of harm then is $\:{p}_{1}\cdot\:{p}_{2}$ [8]. Since our approach focuses on the particular probabilities for $\:FP$ and $\:FN$, e.g. $\:P\left(FN\right)=\:P\cdot\:FNR\left(s\right)$, i.e. the hazards, this refers to the probability $\:{p}_{1}$. Thus, the probability $\:{p}_{2}$ has to be integrated into the weight factors $\:{w}_{FP}$ and $\:{w}_{FN}$, when considering the expected risk $\:ER\left(s\right)={w}_{FP}\cdot\:N\cdot\:FPR\left(s\right)+{w}_{FN}\cdot\:P\cdot\:FNR\left(s\right).$ Additionally, there may be other measures, e.g. other tests or effective therapies also in later stages, which could have the potential to mitigate the risk in terms of probability or severity. These would also have to be integrated into the weights $\:{w}_{FP}$ and $\:{w}_{FN}$. Even though such options were not elaborated in this paper, they can be addressed appropriately, using our approach.

#### Application of the identified regulatory requirements to the concrete use scenarios

After clarifying the relevant regulatory requirements and their relation to the risk-based assessment approach, we checked how these results apply to the use scenarios provided in Research Question C – Integration into the development process for ML-based medical devices. These scenarios include substantial differences in the risk profiles. The analysis for each particular scenario can be found in Table 6. Mind that all of these use scenarios were designed in a way, that the probabilities for the different types or errors / risks were assumed to be equal. Only the severities / costs for the risks and subsequently the overall risk ratios differed. Additionally, a default risk ratio of $\:{c}_{FN}=1$ was assumed for the reference scenario considered as a case of moderate risk. Within this analysis, the deviations of the risk ratio according to the reported risk aspects were roughly estimated, since it was only intended to demonstrate the tendency of the effects.

**Table 6:** Results for use scenarios. Analysis of use scenarios as introduced in Research Question C – Integration into the development process for ML-based medical devices: impact of the characteristics of the particular use cases onto the overall risk ratio. A default risk ratio of $\:{c}_{FN}=1$ was assumed as a reference for moderate risk levels. The deviations to this default value due to the details in the particular case were rated

Use scenario	Implication onto costs / overall risk ratio
A. *diagnostic test*: ML-based system which is integrated into a screening test for a specific disease (e.g. a specific type of cancer). The actual prevalence of the disease as well as the probabilities of different types of errors / risks, i.e.$\:{T}{P}$,$\:{F}{N}$,$\:{T}{N}$, and$\:{F}{P}$, is assumed to be fixed in the following subcases.	
A1. Situation with very high risk in case of false negatives ($\:{F}{N}$), when an early detection of the disease is missed. For example, this can be the case when the disease quickly develops into a critical state where the success rate of potential treatments is very limited	substantially higher costs for$\:FN$ $\:\to\:{c}_{FN}\gg\:1$
A2. Situation still with high risk in case of false negatives ($\:{F}{N}$), but with an option to better detect the disease by additional tests	more moderate costs for$\:FN$, if the test is integrated as an additional measure; impact depends on the quality of the additional test
A3. Situation with reduced risk in case of false negatives ($\:{F}{N}$), because the disease develops rather slowly and has less severe impact	moderate to low costs for$\:FN$ $\:\to\:{c}_{FN}<1$
A4. Situation with reduced risk in case of false negatives ($\:{F}{N}$), like in scenario AA3, but additionally with high risk in the case of false negatives ($\:{F}{P}$). For example, this may be the case, when a biopsy or another treatment needs to be performed in the case of positively predicted case (i.e.$\:{T}{P}$and$\:{F}{P}$). Such additional treatments may also cause substantial harm to the patient.	substantially higher costs for$\:FP$ $\:\to\:{c}_{FN}\ll\:1$ (if not counter-balanced by other types of harm)
B. *quality inspection*: ML-based quality assurance system for identifying deficiencies in surgical instruments before they get delivered. It is assumed that the same ratio relationships is given as in use scenario A. This refers to the relationships between positive (instrument has a defect) and negative cases (instrument has no defect) as well as error cases (i.e.$\:{T}{P}$,$\:{F}{N}$,$\:{T}{N}$, and$\:{F}{P}$).	
B1. Situation where instruments with a missed detection of a defect ($\:{F}{N}$) will be delivered directly to a hospital and may cause serious harm to a patient when applied in the treatment procedure	potentially high costs for$\:FN$, if defect cannot be detected otherwise $\:\to\:{c}_{FN}>1$
B2. Situation as in case BB1, but this time including an additional check in the hospital which substantially lowers the probability and/or severity of the potential harm of$\:{F}{N}$cases	Substantially lower costs for$\:FN$in comparison to scenario BB1 $\:\to\:{c}_{FN}<1$
B3. Situation where the quality assurance step is designed to identify defects in an early production step. The particular instrument is eliminated in this case to reduce further financial costs, caused by$\:{F}{P}$. In this case, it is considered that additional quality steps are included to keep the$\:{F}{N}$rate at an appropriate level, e.g. additional visual inspections or tests, which reduce the risk of delivering defect instruments / producing harm on the patient to a low and acceptable level.	only limited impact on clinical aspects, but the company should be interested to do a cost-based assessment due to financial reasons

As a result, it can be recognized that there are several situations which lead to risk ratios $\:{c}_{FN}$ which may considerably deviate from $\:{c}_{FN}=1$. This includes deviations in either direction, e.g. increases of $\:{c}_{FN}$ due to higher risks for $\:FN$ cases as well as decreases of $\:{c}_{FN}$ due to lower risks for $\:FN$ cases as well as higher risks for $\:FN$ cases. Mind that one step up in the risk matrix usually corresponds with an increase of the risk ratio by a factor of $\:10$, in our scenarios. Additionally, there are cases where the impact depends on other measures (e.g. additional tests or the impact of specific treatment options). In these cases, the chain of effects needs to be considered in order to obtain a proper estimation of the overall risk ratio.

#### Overall applicability and consistency of the developed risk-based approach in relation to the regulatory requirements in the EU

The regulatory requirements in the EU demonstrate that addressing risks is a core topic for the development of ML based devices. This is due to the MDR, ISO 14971, ISO/TR 24971, and the AI Act. Across the entire development and product life cycle, an evaluation is indicated which includes potential risks and benefits, in a systematic way. The presented risk-based approach is consistent with the basic definitions of risk according to the MDR [6], ISO 14971 [8], and ISO/TR 24971 [9]. The probability and severity of risks can be integrated into the weights that are used in our approach. At least, this is the case for scenarios with a limited complexity. However, the approach can also be extended to more complex cases, appropriately. The probabilistic approach naturally aligns with standard descriptions of risk categories and the risk matrix, as e.g. defined in ISO 14971 and ISO/TR 24971. The approach also aligns with the basic requirements in the AI Act [7]. Additionally, the AI Act states in recital 64 that the providers of medical devices “should have flexibility with regard to operational decisions on how to ensure compliance of a product that contains one or more AI systems with all the applicable requirements of that Union harmonised legislation in an optimal manner” [7]. Thus, the basic approach for risk management given in the MDR and ISO 14971 as the respective harmonized standard should also be applicable in ML based medical devices. This is also consistent with BSI/AAMI 34971 [10], since there are no additional requirements in this direction, in this technical report. However, it remains to be shown that finally our approach is consistent with all regulatory requirements in the EU, since harmonized standards are not provided for the AI Act, yet.

## Discussion

Within this paper, we demonstrated the necessity as well as the impact of a risk-based approach for the evaluation of ML-based medical devices, in particular for classification tasks.

### Research question A – utilization of risk-based performance metrics in recent publications

With respect to research question A, we showed that risk-based approaches currently do not play a substantial role in the scientific literature, when assessing the performance of ML-based classification models. Basically, standard metrics like $\:BA$, $\:F1$ score, or $\:MCC$ are applied for this. This was demonstrated by a non-exhaustive literature research for an exemplary time period. According to this study, risk-based aspects are only integrated / reported in a low percentage of papers. When we counted the publications, which addressed risk prediction as the main application, as positive results, we got 3 out of 30 cases, i.e. 10%, with a 95% CI of $\:0.24$, in the best case. When we excluded these cases fully, we got down to 0 out of 27 cases, with a 95% CI of $\:0.11$. In any case, the application of risk-based approaches was very limited and restricted to cases where risk prediction was a main topic itself.

### Research question B – impact of risk factors into performance metrics

With respect to research question B, an approach for integrating risk factors into the evaluation of ML-based classification models was provided. In particular, dedicated weights were integrated for the different types of errors (false positives – $\:FP$ and false negatives – $\:FN$) into the balanced accuracy ($\:BA$) metric as a standard performance measure. This resulted in an evaluation of ML classification models in terms of the expected risk $\:ER$ respectively $\:\stackrel{\sim}{ER}$. It was demonstrated that $\:ER$ is equivalent to a performance metric, which is a weighted version of $\:BA$. Thus, this metric was subsequently called Weighted Balanced Accuracy ($\:WBA$). An artificial error distribution based on modified Gaussian distributions was utilized to analyze the impact of different risk ratios on the resulting overall expected risk. It was demonstrated, that the relative increase with respect to $\:\stackrel{\sim}{ER}$ for the analyzed parameter settings increases up to $\:198\%$ for risk ratios $\:{c}_{FN}$ of $\:0.1$ and $\:10.0$.This represents the case when the weights for the different types of errors $\:FP$ and $\:FN$ differ by such a factor. The term relative increase refers to the situation, when an unweighted threshold selection (i.e. risk ratio $\:{c}_{FN}=\:1$) would have been performed instead of the actual risk ratio. Risk ratios $\:{c}_{FN}$ of $\:0.1$ and $\:10.0$ represent important benchmarks since they typically correspond with an de-/increase of one level in the risk matrix, as they are often applied for medical devices according to [9]. For risk ratios in the range between $\:0.5$ and $\:2.0$, the increase in $\:\stackrel{\sim}{ER}$ remains lower than $\:12\%$, in our example.

### Research question C – integration into the development process for ML-based medical devices

With respect to research question C, the impact of these findings was analyzed in relationship to the regulatory requirements for the development of AI-based medical devices as given by the corresponding regulations and standards. In particular, this referred to the situation in the EU, with the MDR [6] as the main regulation for medical devices and the ISO 14971 [8] as the relevant standard for risk management. This was accompanied by the technical report ISO/TR 24791 [9] as a guidance for applying [8] as well as the proposed AI Act of the EU [7], which probably has to be applied for many AI-based medical devices in the future, in its then final version. It was demonstrated, that a neutral risk profile (with overall risk ratio $\:=1$) basically requires, that the probability and severity of a risk have a reciprocal relationship, i.e. their product equals $\:1$ when using a multiplicative approach for combining severity and probability levels. Since the latter are often given in exponential steps, the severity levels would need to have the same increase to achieve a balanced situation. Using exemplary application scenarios, we demonstrated that deviations from a reference scenario (considered as a neutral case) can occur in either direction. Since an increase of the risk ratio by a factor $\:10$ typically refers to an increase of one level in the risk matrix, the range of risk ratios used in this paper are considered to represent reasonable scenarios for such applications. Thus, a risk-based evaluation of AI-based medical devices is required by the regulations and standards and needs to be considered in the definition of appropriate, use-case specific performance metrics. We also demonstrated that our approach addresses the requirements from the regulations in the EU, in a consistent way. This includes the MDR [6] as well as ISO 14971 [8] and ISO/TR 24791 [9]. For the AI Act [7], there is also alignment with the basic requirements. However, this cannot finally be judged at the moment, since the harmonized standards for the AI Act are not available yet.

### Summary of research questions

In summary, we see that there is a discrepancy between the regulatory requirements and the presentation of results in scientific papers. It may be considered an open question whether scientific papers should also include risk-based considerations. On the one hand, they often represent feasibility studies for developing suited ML models. Thus, they may be placed outside an actual product lifecycle. On the other hand, the development of these models gets part of this lifecycle when the models are used in concrete applications. This step is strongly connected to the development process and has interdependencies with risks and clinical impact. For this reason, the regulations, e.g. MDR, AI Act, and ISO 14971, require to apply / consider risk management activities, in the entire development process. From our perspective, the clinical impact should be included as a basic objective in scientific papers in the field of ML-based medical applications. Otherwise, the validity of the methods remains unclear and the translation into clinical practice difficult. An assessment using standard error / accuracy metrics that do not integrate benefits and risks of the device remains deficient. Such an approach does not sufficiently address the actual impact of the developed model, in the particular clinical application.

It may be asked whether a risk-based assessment is strictly required for the development of ML models. If we follow the requirements in the regulations consequently, we have to include such an approach as long as the integration of risk factors substantially changes the risk-benefit relationship [6]. As described, this usually is triggered by a step-up in the risk matrix (in the diagonal direction). At least, this is the case when the corresponding risk is in the critical area (i.e. between acceptable and not acceptable). It needs to be mentioned, that the reduction of risks does not necessarily be performed by an integration of risk-based factors in the training, validation and testing phases. For example, it could also be implemented by adjusting threshold parameters according to a risk-based approach, when the model provides such threshold parameters and when this approach achieves an equivalent reduction of risks. But at the end, the performance of the model / medical device needs to be described in terms of the clinical impact, in particular with respect to the risk-benefit relationship.

It may be argued that current practices usually do not include risk-based assessments, as this was demonstrated according to the results for research question A. Additionally, neither the current regulations (MDR, AI Act) nor the corresponding standards (ISO 14971, ISO/TR 24971, BSI/AAMI 34971) do explicitly describe / require such a risk-based approach. In general, standards are considered as state-of-the-art for the implementation of regulatory requirements. In particular, this is the case (in the EU) when they represent harmonized standards [6, 7]. Detrimentally, ISO 14971 and ISO/TR 24971 do not provide specific guidance for ML-based devices. BSI/AAMI 34971 goes into this direction, but does not contribute to core elements of the risk management process which are relevant, in our paper.

The AI Act and its corresponding standards are intended to fill parts of this gap, on a horizontal level, i.e. for all sectors and not with a focus on medical applications. Additionally, the development of harmonized standards for the AI Act has only started recently, in correspondence with the legislative process of the AI Act. Thus, there is only limited guidance in this direction as well. Subsequently, there is no accepted state-of-the-art. This means, that developers of ML-based models / devices have to justify themselves how they meet the regulatory requirements.

Additionally, we point out that there are some further limitations regarding the application of risk-based approaches for the assessment of ML-based medical devices. One critical aspect in this process is the question how to get to appropriate probabilities and costs for the particular risks. If they are known, they should be integrated into the evaluation of the ML-based models according to the discussed requirements in the MDR [6] and risk management standard [8]. If they are not known, the question is whether and to what detail they actively need to be determined during the development phase. This may depend on the particular use case and thus, needs to be analyzed on this level. As an alternative, it may be possible or required to collect data during the operation period of the device, within the post market surveillance activities. Thus, an incremental strategy for the more detailed determination of risk factors may be feasible.

In general, risk management should be considered and implemented as a continuing process. According to the MDR [6] as well as the proposed AI Act [7], it is also necessary to thoroughly follow up the results of the operation phase and eventually update the device, if the risk profile substantially changes. As already mentioned, it is allowed to perform a semi-quantitative or even qualitative assessment of the risks, according to [8, 9]. This allows that certain levels of risk can be grouped together and categorized with respect to the probability as well as the severity level. This renders the assessment of risks more practicable.

The proposed approach helps to implement a risk-based assessment of ML models for classification in the field of medical devices. It is consistent with existing regulations and standards in the EU for medical devices. There still is a gap with respect to the AI Act, since no harmonized standards are available yet. For providers of ML-based systems, provision of appropriate standards to clarify the regulatory requirements is paramount. In the EU, harmonized standards by definition are central elements to achieve this. Regarding risk management, it will be crucial that the requirements in the sectoral and horizontal regulations, i.e. MDR and AI Act, are finally aligned. Currently, there are discrepancies which need to be sorted out. Otherwise, considerable obstacles would remain for ML-based medical devices. Additionally, there currently are no clear guidelines or standards how to implement a risk-based approach for the evaluation of ML models. Often the consideration of risk-based elements seems to be avoided, even though it would be required from a regulatory perspective. At least, this applies to a substantial amount of scientific publications in this field, as it was demonstrated in research question A. The approach presented in this paper provides a proposal for this implementation. It provides basic ingredients, which can be integrated into the entire development and product life cycle of an ML-based medical device. The methods may need to be adapted to particular use cases according to the complexity and knowledge about the risks, which are relevant for the particular device.

### Relation to existing approaches

In the literature, there already are some approaches to include costs and benefits into the evaluation of ML-based classification tasks as discussed in the introduction, see e.g [13, 14, 17–23, 60]. Some of them apply to AI in general, some of them focus in medical applications. The approach presented in this paper utilizes basic aspects of this methodology, in particular within the framework of normative decision theory, and applies it to the risk-based development of medical devices. It substantially extends the preliminary results provided in [24].

In decision / utility theory, additional methods have been developed which address more sophisticated types of decisions. For example, deeper chains of events in the decision process can be addressed e.g. using influence diagrams [17]. This allows to implement cascades of risk measures or sequences starting from hazardous situations to harms and finally to risks. Additionally, decision theory provides options to include non-linear ratings which e.g. represent a stronger risk averse behavior, i.e. over-proportionately avoid risks. In particular, such extensions can be applied to deal with situations where combined risk values are not calculated by a multiplicative approach but another type of combination [17]. Further on, the impact of uncertainties, e.g. in terms of uncertainty aversion, as well as their treatment, e.g. using the value of information approach, can be utilized [17]. This could e.g. be used to include the detectability of specific errors and risks in the calculation. This could also address the potential costs to obtain further valuable information, e.g. about a certain disease or therapy using additional diagnostic tests.

Even though such factors are not included in this paper, our basic approach can be extended into this direction in future steps. Basically, it is compatible with the methodology of decision theory. However, the proposed methodology provides components for the integration of risk factors into the evaluation of ML-based classification models. Based on this, important regulatory requirements can be addressed as given in [8].

The utilization of application-specific risk factors also has some challenges. First of all, the reliable assessment of probabilities and the definition of appropriate costs / weights for the different risks can be problematic. In particular, it often has to be defined how serious / critical harms should be balanced with other types of impact, e.g. additional personal burdens or costs. For balancing critical harms or even deaths with costs, the quality-adjusted life years (QALY) approach can be utilized. It basically relates to the question how much money persons are willing to spend to reach or maintain a certain level of health [22, 61]. These costs have to be coupled with the probabilities, which are also often unknown during development. Another option is the usage of micromorts. It is based on the question how much a person is willing to accept for a lottery representing a death probability of 1 in a million [23, 62]. 

To integrate risk factors into the development of products, the standard for risk management for medical devices ISO 14971 [8] allows some pragmatic simplifications. On the one hand, the probabilities may be clustered in a semi-quantitative or even qualitative way based on estimations by experts. On the other hand, the risk assessment can / should be updated after its placement on the market according to systematically acquired data from operation phase. When both factors, i.e. probabilities and costs / severity, are available, the product of these two factors provides the combined risk ratio. This reciprocal relationship was graphically shown in Fig. 6. In terms of decision theory, the different levels of risk ratio represent a so-called preference relationship (see [17] for basic definition of preference relations). Such relationships are crucial to define situations when different parameters, i.e. different aspects of utility or costs, are balanced out. In our case, this constitutes in which situations the particular risks, e.g. risks caused by $\:FP$ vs. $\:FN$ cases, are balanced out. They are constituted by the iso-level lines of the preference relationship. Again, this builds a bridge between our approach and the methodology developed in decision theory.

Using application-specific performance metrics has some other limitations. The comparability of different scientific approaches or models gets more challenging. Standardized metrics have the advantage that the models can be rated according to a generally established method as emphasized e.g. in [12]. Additionally, standardized metrics are examined in more detail and thus, may reflect a higher level of interpretability. This may be increased when risk-based assessment methods include multiple factors and get more complex. But, standard metrics may also achieve a lower interpretability. Values like specificity, sensitivity, $\:F1$ score, $\:MCC$ are abstract numbers which are hard to understand for many people. In particular this may apply to doctors or patients which are part of the intended population for the medical device. A risk-based approach better describes the results in terms of clinical, application-specific outcomes. This provides better access to the actual use of a model, including its risks / costs as well as its benefits.

### Limitations of the study

The study / methods used in this paper have some limitations. First, the analysis of scientific literature was only performed for an exemplary period of time. It does not reflect the entire state-of-the-art which risk-based approaches already were developed and how often they were applied. Second, we only used an artificial model for the error distributions in our analysis and not results from a model which comes from a real-world scenario with an actually trained ML model. This includes, that our model is continuous and also differentiable, which makes it easier to align the tangents of the $\:ROC$ curve with the iso-contours of the metric. We also focused on symmetrical models for most of the analysis steps. Thus, it makes sense to apply our approach in real-world scenarios. Third, the current approach was focused on relatively simple decision cases. For example, it does not include the costs for the correctly assigned cases. Additionally, it does not present cases where the decision has to follow a deeper structure of decisions. For example, this could refer to different probabilities and severities of developing a serious disease in the case of missed diagnosis, i.e. $\:FN$ cases. Another potential component could be the integration of risk mitigation measure, like performing additional tests to safeguard a diagnosis or other measures to reduce the impact of a missed diagnosis. This would have to be addressed in deeper levels of the probabilistic decision structure according to the options already provided by decision theory [17]. 

## Conclusion

The aim of this paper was not to provide a comprehensive methodology for implementing an extensive set of decisions. It was considered as a starting point to better address a more application-specific and value-based approach, which includes actual clinical factors like associated risks into the evaluation of ML-based medical devices. Thus, it wants to create awareness towards a more risk-based way of measuring performance, with a focus on ML-based classification tasks. Based on the results of this paper, it can be recognized that a systematic integration of risk factors into the evaluation of AI-based medical devices is necessary – from a regulatory perspective as well as for an application-specific optimization of clinical outcomes. The paper demonstrates that risk factors are currently only considered in a low percentage of scientific publications. In order to better address an assessment based on the clinical impact of the device, this paper provides a basic methodology to systematically integrate risk factors into the evaluation of ML-based classification models. It demonstrates that this approach is in compliance with current and upcoming regulatory requirements for their use in medical devices. The paper also wants to contribute to the discussions about appropriate guidelines of regulatory requirements, including the preparation and provision of corresponding (harmonized) standards.

## Supplementary Information

Supplementary Material 1.

## Data Availability

The study was performed based on artificial models and no actual data was used. The detailed outcome data are available from the corresponding author on reasonable request.

## Abbreviations

AI: Artificial intelligence
ML: Machine learning
MDR: Medical device regulation
EU: European Union
TP: True positives
FP: False positives
TN: True negatives
FN: False negatives
FPR: False positive rate
FNR: False negative rate
Acc: Accuracy
BA: Balanced accuracy
Prec: Precision
Sen: Sensitivity
Spec: Specificity
NPV: Negative predictive value
PPV: Positive predictive value
F1: F1 score
MCC: Matthews correlation coefficient
ROC: Receiver operating characteristics
AUROC: Area under the ROC Curve
PRC: Precision-recall curve
WBA: Weighted balanced accuracy
ER: Expected risk
