# Supplementary material for: Risk-based evaluation of machine learning-based classification methods used for medical devices
Source: BMC Med Inform Decis Mak. 2025 Mar 11;25:126. doi: 10.1186/s12911-025-02909-9 (PMC11895222; doi:10.1186/s12911-025-02909-9)
Supplement: Supplementary file 1 — Supplementary Material 1. [file 12911_2025_2909_MOESM1_ESM.pdf]

Table S1. List of articles which were excluded in the literature research regarding recent publications about performance metrics of ML-based classification models (additional information to section **Error! Reference source not found.**). The table documents the used performance the reason for exclusion.

Publication (bibliographic information) + reason for exclusion
Kamran M, Ullah B, Ahmad M et al. (2022) Application of KNN-based isometric mapping and fuzzy c-means algorithm to predict short-term rockburst risk in deep underground projects. Front Public Health 10:1023890. https://doi.org/10.3389/fpubh.2022.1023890<br>**Reason for exclusion - observer 1:** not a medical application<br>**Reason for exclusion - observer 2:** urban engineering; not medical
Wang WK, Chen I, Hershkovich L et al. (2022) A Systematic Review of Time Series Classification Techniques Used in Biomedical Applications. Sensors (Basel) 22. https://doi.org/10.3390/s22208016<br>**Reason for exclusion - observer 1:** only review<br>**Reason for exclusion - observer 2:** only review paper
Abbasi A, Javed AR, Iqbal F et al. (2022) Deep learning for religious and continent-based toxic content detection and classification. Sci Rep 12:17478. https://doi.org/10.1038/s41598-022-22523-3<br>**Reason for exclusion - observer 1:** not a medical application<br>**Reason for exclusion - observer 2:** toxic language: no medical
Konings D, Alam F, Faulkner N et al. (2022) Identity and Gender Recognition Using a Capacitive Sensing Floor and Neural Networks. Sensors (Basel) 22. https://doi.org/10.3390/s22197206<br>**Reason for exclusion - observer 1:** not really a medical application, only gender classification according to walking characteristics<br>**Reason for exclusion - observer 2:** only gender classification
Olthof AW, van Ooijen PM, Cornelissen LJ (2022) The natural language processing of radiology requests and reports of chest imaging: Comparing five transformer models' multilabel classification and a proof-of-concept study. Health Informatics J 28:14604582221131198. https://doi.org/10.1177/14604582221131198<br>**Reason for exclusion - observer 1:** not a classification with a low number of classes, no risk assessment included<br>**Reason for exclusion - observer 2:** NLP; not classification
Stoitsas K, Bahulikar S, Munter L de et al. (2022) Clustering of trauma patients based on longitudinal data and the application of machine learning to predict recovery. Sci Rep 12:16990. https://doi.org/10.1038/s41598-022-21390-2<br>**Reason for exclusion - observer 1:** clustering and no classification task<br>**Reason for exclusion - observer 2:** initially included by observer 2, but agreed that clustering as a form of unsupervised learning is not classification as a form of supervised learning and thus the paper should be excluded
Bibi R, Mehmood Z, Munshi A et al. (2022) Deep features optimization based on a transfer learning, genetic algorithm, and extreme learning machine for robust content-based image retrieval. PLoS One 17:e0274764. https://doi.org/10.1371/journal.pone.0274764

**Reason for exclusion - observer 1:** general image classification models applied to some test data sets, no dedicated medical study / application<br>**Reason for exclusion - observer 2:** no medical association
Ferreira-Santos D, Amorim P, Silva Martins T et al. (2022) Enabling Early Obstructive Sleep Apnea Diagnosis With Machine Learning: Systematic Review. J Med Internet Res 24:e39452. https://doi.org/10.2196/39452<br>**Reason for exclusion - observer 1: only review**<br>**Reason for exclusion - observer 2:** only review paper
Zainab K, Srivastava G, Mago V (2022) Identifying health related occupations of Twitter users through word embedding and deep neural networks. BMC Bioinformatics 22:630. https://doi.org/10.1186/s12859-022-04933-2<br>**Reason for exclusion - observer 1:** not directly a medical application, only identification of health occupation in twitter messages<br>**Reason for exclusion - observer 2:** no medical association
Carissimo C, Cerro G, Ferrigno L et al. (2022) Development and Assessment of a Movement Disorder Simulator Based on Inertial Data. Sensors (Basel) 22. https://doi.org/10.3390/s22176341<br>**Reason for exclusion - observer 1:** no study with patients involved, only a simulator was used for assessing movement disorders<br>**Reason for exclusion - observer 2:** simulation
Ucer S, Ozyer T, Alhajj R (2022) Explainable artificial intelligence through graph theory by generalized social network analysis-based classifier. Sci Rep 12:15210. https://doi.org/10.1038/s41598-022-19419-7<br>**Reason for exclusion - observer 1:** not an actual medical study<br>**Reason for exclusion - observer 2:** no medical association
Wang C, Li C, Zhang R et al. (2022) Identification of radiographic characteristics associated with pain in hallux valgus patients: A preliminary machine learning study. Front Public Health 10:943026. https://doi.org/10.3389/fpubh.2022.943026<br>**Reason for exclusion - observer 1:** binary classification was not a main focus<br>**Reason for exclusion - observer 2:** initially included by the observer 2, but agreed during the discussion that this is an NLP application where binary classification with low number of classes was not the main focus and thus, the paper should be excluded
Goodman-Meza D, Shover CL, Medina JA et al. (2022) Development and Validation of Machine Models Using Natural Language Processing to Classify Substances Involved in Overdose Deaths. JAMA Netw Open 5:e2225593. https://doi.org/10.1001/jamanetworkopen.2022.25593<br>**Reason for exclusion - observer 1:** not an actual medical task: extraction of reasons of death using NLP applied to death certificates, binary classification with low number of classes was not the main focus<br>**Reason for exclusion - observer 2:** no medical association
Bockelmann N, Schetelig D, Kesslau D et al. (2022) Toward intraoperative tissue classification: exploiting signal feedback from an ultrasonic aspirator for brain tissue differentiation. Int J Comput Assist Radiol Surg 17:1591–1599. https://doi.org/10.1007/s11548-022-02713-0<br>**Reason for exclusion - observer 1:** not an actual patient study, only simulated material

**Reason for exclusion - observer 2:** initially included by the observer 2, but agreed during the discussion that this is only a simulation and not an actual patient study and thus, the paper should be excluded
Belue MJ, Turkbey B (2022) Tasks for artificial intelligence in prostate MRI. Eur Radiol Exp 6:33. https://doi.org/10.1186/s41747-022-00287-9<br>**Reason for exclusion - observer 1:** only narrative review<br>**Reason for exclusion - observer 2:** only review paper
Suresh K, Severn C, Ghosh D (2022) Survival prediction models: an introduction to discrete-time modeling. BMC Med Res Methodol 22:207. https://doi.org/10.1186/s12874-022-01679-6<br>**Reason for exclusion - observer 1:** not a concrete study, but only exemplary, publicly available data sets, binary classification not the main focus, basic methodology as the main focus<br>**Reason for exclusion - observer 2:** no classification;
Carré A, Battistella E, Niyoteka S et al. (2022) AutoComBat: a generic method for harmonizing MRI-based radiomic features. Sci Rep 12:12762. https://doi.org/10.1038/s41598-022-16609-1<br>**Reason for exclusion - observer 1:** binary classification with low number of classes was not the main focus<br>**Reason for exclusion - observer 2:** initially included by the observer 2, but agreed during the discussion that this is an NLP application where binary classification with low number of classes was not the main focus and thus, the paper should be excluded
Flores-Munguía C, Ortiz-Bayliss JC, Terashima-Marín H (2022) Leveraging a Neuroevolutionary Approach for Classifying Violent Behavior in Video. Comput Intell Neurosci 2022:1279945. https://doi.org/10.1155/2022/1279945<br>**Reason for exclusion - observer 1:** not a medical application, only video surveillance included without a medical task<br>**Reason for exclusion - observer 2:** no medical
Ktistakis E, Skaramagkas V, Manousos D et al. (2022) COLET: A dataset for COgnitive workLoad estimation based on eye-tracking. Comput Methods Programs Biomed 224:106989. https://doi.org/10.1016/j.cmpb.2022.106989<br>**Reason for exclusion - observer 1:** not a direct medical application, only workload estimation, focus not on binary classification<br>**Reason for exclusion - observer 2:** classification of cognitive workload levels solely based on eye data
Wang S, Tang L, Majety A et al. (2022) Trustworthy assertion classification through prompting. J Biomed Inform 132:104139. https://doi.org/10.1016/j.jbi.2022.104139<br>**Reason for exclusion - observer 1:** NLP application -> simple binary classification not a main focus<br>**Reason for exclusion - observer 2:** NLP; not classification
Site A, Vasudevan S, Afolaranmi SO et al. (2022) A Machine-Learning-Based Analysis of the Relationships between Loneliness Metrics and Mobility Patterns for Elderly. Sensors (Basel) 22. https://doi.org/10.3390/s22134946<br>**Reason for exclusion - observer 1:** not a direct medical application, only detection of loneliness of persons, main focus not on binary classification<br>**Reason for exclusion - observer 2:** loneliness; not medical

Li X, Peng D, Wang Y (2022) Improving patient self-description in Chinese online consultation using contextual prompts. BMC Med Inform Decis Mak 22:170. https://doi.org/10.1186/s12911-022-01909-3

Reason for exclusion - observer 1: focus not on classification with a low number of classes

Reason for exclusion - observer 2: NLP; not classification

Sarwar MU, Gillani LF, Almadhor A et al. (2022) Improving Recognition of Overlapping Activities with Less Interclass Variations in Smart Homes through Clustering-Based Classification. Comput Intell Neurosci 2022:8303856. https://doi.org/10.1155/2022/8303856

Reason for exclusion - observer 1: not a direct medical application, only recognition of activities in a smart home environment, focus not on classification with a low number of classes, semi-supervised technique (clustering + classification) in a complex scenario

Reason for exclusion - observer 2: healthcare in smart home: not medical

Sanchis-Segura C, Aguirre N, Cruz-Gómez ÁJ et al. (2022) Beyond "sex prediction": Estimating and interpreting multivariate sex differences and similarities in the brain. Neuroimage 257:119343. https://doi.org/10.1016/j.neuroimage.2022.119343

Reason for exclusion - observer 1: not a direct medical application, only gender discrimination

Reason for exclusion - observer 2: gender classification: not medical

Shah SA, Nwaru BI, Sheikh A et al. (2022) Development and validation of a multivariable mortality risk prediction model for COPD in primary care. NPJ Prim Care Respir Med 32:21. https://doi.org/10.1038/s41533-022-00280-0

Reason for exclusion - observer 1: binary classification was not the main focus

Reason for exclusion - observer 2: initially included by the observer 2, but agreed during the discussion that regression and not classification was the addressed task. Thus, the paper should be excluded.

Aldhyani THH, Alsubari SN, Alshebami AS, Alkahtani H, Ahmed ZAT. Detecting and Analyzing Suicidal Ideation on Social Media Using Deep Learning and Machine Learning Models. Int J Environ Res Public Health. 2022 Oct 3;19(19):12635. doi: 10.3390/ijerph191912635.

Reason for exclusion - observer 1: initially included by the observer 1, but agreed during the discussion that this is an NLP-based application for suicide detection according to social media and not a dedicated medical task. Thus, the paper should be excluded.

Reason for exclusion - observer 2: suicide detection: no medical
